# Calibrated Variant Effect Prediction at the Residue Level Using Conditional Score Distributions

**DOI:** 10.1101/2025.11.24.690189

**Published:** 2025-11-26

**Authors:** Gal Passi, Sapir Amittai, Dina Schneidman-Duhovny

**Affiliations:** 1 The Rachel and Selim Benin School of Computer Science and Engineering, The Hebrew University of Jerusalem, Jerusalem, Israel

## Abstract

Effective clinical use of variant effect prediction (VEP) requires models that are both accurate and well-calibrated. Calibration refers to a model’s ability to produce meaningful and reliable probability estimates. Here, we propose a practical path toward robust VEP calibration by calibrating at the residue-level rather than using global or per-protein schemes. We identify variant subgroups that benefit from targeted calibration and show that, while VEPs appear well calibrated on average, they remain markedly miscalibrated within these subgroups. Leveraging these insights, we develop RaCoon (Residue-aware Calibration via Conditional distributions), implemented on ESM1b, which provides multicalibrated and interpretable predictions across diverse variant subgroups and significantly improves performance across multiple benchmarks. Targeted residue-level calibration not only improves overall calibration but, for most models, also yields gains in global AUROC. Specifically, RaCoon increases AUCROC from 0.912 to 0.924. Our calibration strategy, guided by model-specific feature distributions, is readily transferable to other VEPs.

Accurate variant effect prediction (VEP) is central to protein design, medical genomics, and variant forecasting. Recent advances in deep learning have enabled models that effectively capture evolutionary patterns from protein sequences and structures^1–6^, achieving groundbreaking accuracy in VEP. These models can be broadly categorized into supervised, unsupervised, and weakly supervised approaches.

VEPs are typically evaluated using datasets of clinically reported genetic variants, such as ClinVar^7^, or experimental benchmarks such as ProteinGym, which aggregates data from multiple deep mutational scans (DMS) assays^8^. Two broadly adopted metrics to assess VEP performance are the global AUROC, which measures ranking performance across all proteins, and per-protein AUROC, which captures discriminative ability within individual proteins. State-of-the-art (SOTA) models achieve AUROCs as high as 0.92–0.93 (unsupervised) to 0.98 (supervised) on the ProteinGym and on a ClinVar-derived benchmarks^9^.

While supervised approaches^10–13^ achieve impressive accuracy in specific proteins or families, they are sensitive to sparse and biased clinical annotations and generalize poorly to unseen protein regions. Consistently, AUROC is strongly driven by protein features^14,15^, using the per-protein label ratio alone on ClinVar was shown to achieve an AUROC of 0.914^6,16,17^. Notably, in ClinVar, only ~4% of genes have more than five labeled pathogenic and benign variants. These biases also extend to experimental datasets, where VEP performance varies with assay type, protein context, and data depth^18,19^.

These limitations have motivated the growing use of unsupervised VEPs, less reliant on labeled data and therefore less affected by annotation bias. Unsupervised VEPs can be broadly categorized into alignment-based approaches, trained on multiple sequence alignments (MSAs)^1,20^, protein language models (PLMs) trained on unaligned protein sequences^4,21–24^, and hybrid approaches^4,21–23,25,26^. While alignment-based approaches perform well, they are limited in regions with shallow alignments, such as intrinsically disordered regions (IDRs) or proteins with few homologs. PLMs can address these gaps, with hybrid retrieval-augmented models further boosting performance by selectively incorporating MSA information. While not exposed to labeled data, unsupervised VEPs still embed assumptions that fail in certain variant subgroups^19,27,28^. To address these biases, recent works employ forms of weak supervision, calibrating scores to specific datasets^6,29,30^, proteins^1,31,32^, diseases^33^ or organisms^34^ at inference time using a subset of labeled data from the relevant domain.

Classifier calibration is defined as a model’s ability to provide meaningful probability estimates^35^. For example, an assigned score of 0.8 should correspond to ~80% of similar variants being pathogenic. However, large-scale models often exhibit poor calibration^36^, and performance frequently degrades on out-of-domain data^37^, such as VEPs evaluated on test sets not overlapping with ClinVar^9^. Calibration is not always correlated with accuracy^38^ and oftentimes, training objectives intended to boost accuracy can reduce calibration^39^.

Several examples illustrate this discordant relationship between accuracy and calibration in VEPs, notably variants in IDRs. IDRs lack stable structure, evolve rapidly, and are more tolerant to mutations^40–43^. Although IDRs make up approximately 27% of the human proteome^44,45^, only 11% of ClinVar pathogenic variants occur in these regions, resulting in a distribution highly skewed toward benign variants. Consequently, variants in IDRs can inflate AUROC through confident benign predictions, reducing sensitivity to pathogenic variants and degrading calibration^14,28,42^. The opposite is observed in N-terminal methionine variants^42^ where ~95% are pathogenic in ClinVar, similarly causing miscalibration. Other examples include variants with distinct physico-chemical properties or few homologs^29,46^. Together, these underscore the need for explicit residue subgroup calibration.

As VEPs reach unprecedented accuracy^15^ and wider clinical use, identifying and calibrating for distribution shifts has gained increasing attention^29,47–49^ While models appear globally calibrated, they may be systematically miscalibrated for specific subgroups, posing challenges for clinical decision making^50^. A multicalibrated model, whose predicted probabilities remain accurate not only overall but also within each relevant subgroup of variants^51^, would therefore be highly desirable for VEPs. Recent studies suggest that multicalibration can often be achieved with minimal post-hoc adjustment^52^ and typically does not compromise, and may even improve, discriminatory performance^51,53^.

Here, we identify multiple subgroup-specific distribution shifts and adjust for them to design multicalibrated VEPs with minimal supervision. We highlight limitations in current VEPs calibration and propose a set of residue attributes that benefit from targeted calibration. Our proposed inference-time calibration approach not only closes existing reliability gaps but also increases AUROC across multiple models. Finally, we introduce RaCoon (Residue-aware Calibration of conditional distributions), a calibrated PLM based on ESM1b, capable of producing interpretable and calibrated pathogenicity predictions and substantially improving the baseline model’s AUROC^4^.

## Results

### Protein-level aggregation masks label shifts captured at the residue-level

A well-established source of heterogeneity in missense datasets is label shift (prior shift^54–56^), where the proportion of pathogenic variants $P\left(Y|M_{i}\right)$ differs across specific attributes $M_{i}$ such as disorder level^42,57,58^, physicochemical properties^59^, and solvent accessibility^60^. Although these prior shifts are highly susceptible to annotation biases, they can still carry meaningful biological signals.

A standard approach to detect prior shifts in missense variant datasets is to compare the pathogenic variant rate within specific subgroups to the pooled rate across the entire dataset. Distribution shifts can be detected at broader protein or family scales^61–64^, the standard practice for calibrating VEPs^1,6,65^. While protein-level calibration captures latent feature combinations without explicit annotation, it can obscure strong residue-level signals and even introduce miscalibration for specific residue types, as shown below.

**Figure 1:**
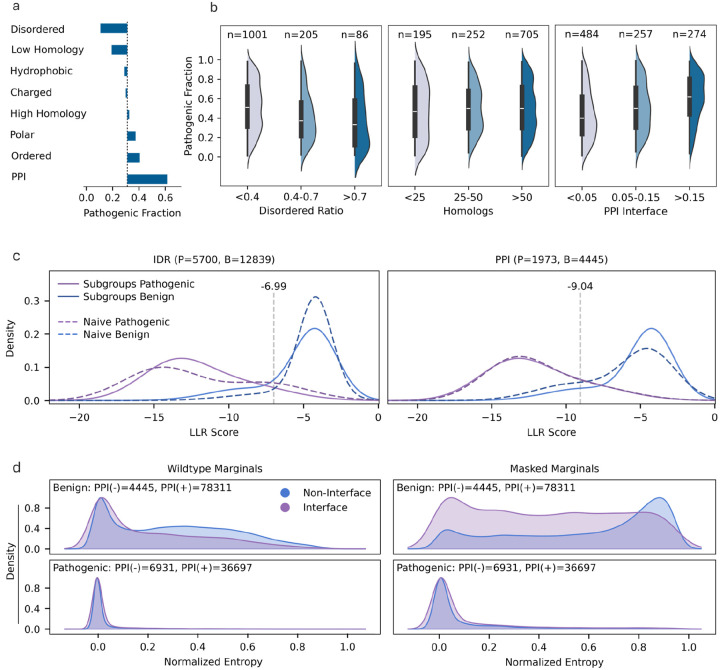
Prior and feature conditional distribution shifts. **a.** Residue-level pathogenic fraction across attributes. The dotted line represents pathogenic variants ratio across the entire ClinVar_HQ dataset (0.29) **b.** Protein-level pathogenic ratio distribution as a function of attribute enrichment in 1,285 well-annotated ClinVar transcripts. **c.** Distribution of LLR scores for pathogenic and benign variants in IDRs (left) and in PPIs (right) vs. naive in the ClinVar_HQ dataset. P,B denote the number of pathogenic and benign variants per subgroup. Dotted line - optimal classification thresholds (J-statistic) Distributions fitted using two-component GMMs. **d.** Conditional distributions of the flipped normalized entropy (1 = complete uncertainty, 0 = complete certainty) for wild-type (left) and masked (right) sequences, grouped by benign (top) and pathogenic (bottom) variants.

We observe significant fluctuations in residue-level pathogenic rates both when examining high-quality clinical labels derived from ClinVar (ClinVar_HQ; N=171,196, Methods) and in experimental benchmarks from ProteinGym (Figure 1a, Table 1). We focus on attributes that can be directly inferred from protein sequence and are well-established in the literature (Methods, Table 4). Pathogenic variants are notably depleted in disordered regions and in sequences with few homologs, but enriched in ordered regions, polar residues, and residues in protein-protein interfaces (PPIs).

To test how residue-level shifts are reflected at the protein level, we curate a set of well-annotated ClinVar proteins (N = 1,285, Methods) with at least ten labeled variants, including at least four pathogenic and four benign. Proteins are grouped by attributes showing the strongest residue-level prior shifts: the proportion of disordered sequence, proportion of residues predicted to participate in PPIs, and the number of homologs. While similar general trends are captured at the protein level, the variation within each group is very wide(Figure 1b). The mutual information (MI) between disorder-level and pathogenicity is markedly higher at the residue level (0.050 ± 0.00) than at the protein level $\left(0.014\pm 0.01,p\ll 1.0e^{-23}\right)$. Although MI differences for homology and PPIs are not statistically significant, protein-level estimates show an order of magnitude higher variability (Table 5, Methods), indicating substantially less stable and informative signal at the protein level.

### Conditional feature distribution shifts separate biological signal from annotation bias

However, considering only the conditioned prior distribution P(Y∣Mi) offers a limited view, prone to annotation biases. In contrast, examining the class-conditional feature distributions, P(X∣Mi), provides a complementary insight into the origins and implications of prior shifts. Prior shifts driven by genuine biological differences, rather than labeling biases, are expected to be reflected in the feature distribution^55,66–68^, particularly in PLMs trained without clinical labels. Conversely, label shifts that are not reflected in these distributions may stem from annotation bias or from limited model capacity to capture the underlying biological dependencies. To study class-conditional feature distributions, we examine ESM1b log-likelihood ratios (LLRs) between wild-type and mutant scores using wild-type marginals^4,21^ (Methods).

We model the baseline ClinVar_HQ scores distribution over the entire dataset by fitting two Gaussian Mixture Models (GMMs)^69^, one for benign and one for pathogenic variants. Subgroup-specific GMMs are then fitted per residue attribute and compared to the baseline to reveal class-conditional feature distribution shifts. To control for prior shifts between subgroups we sample pathogenic and benign variants using the baseline dataset ratio (0.31). Class-conditional LLR-score shifts are most pronounced for disordered and hydrophobic residues, moderate for residues in PPIs and ordered regions, and minimal for polar residues and for residues in proteins with few homologs (Figure 1c, S1). Notably, PPI residues exhibit the strongest label shift but only modest shifts in scores distribution, underscoring the difficulty in calibrating for the corresponding label shifts. Similar results are observed on the ProteinGym^8^ clinical substitution benchmark (Figure S1).

Label shifts in disordered regions reflect their distinct mutational tolerance^42,43^ and are well captured by ESM1b. Conversely, the limited distribution shifts in sequences with few homologs stems from the difficulty in generalizing on these sequences and potential annotation biases. Similarly, while PPI residues are enriched for pathogenic variants^70,71^, the modest distribution shift suggests that ESM1b has limited capacity to capture these features. This interpretation aligns with recent benchmarking studies and with SOTA PPI predictors that augment sequence-based representations with structural or contextual information^72–74^.

While LLR-scores capture class-conditional scores distribution differences, they represent only the wildtype and mutant positions, offering a narrow view of the model’s sensitivity to specific attributes. To broaden the scope, we examine entropy distributions across the entire representation vector, by comparing conditional feature distributions from two complementary attributes $M_{i}$ and $\neg M_{i}$ (for instance, polar vs. non-polar residues). Using different representation strategies: wildtype, mutant, and masked sequences (Methods), we assess whether they provide any advantage in capturing the attribute in question (Figure 1d, Figure S2). For example, variants in PPI residues display only marginal differences using wildtype sequences, whereas stark contrasts emerge with masked sequences. Likewise, distribution shifts between disordered and ordered residues, as well as between polar and non-polar residues, become more pronounced under masked representation (Figure S2). Although the effect is more pronounced for benign variants, shifts are also evident in pathogenic variants, for example, between hydrophobic and hydrophilic residues. Previous works found masked marginals offered little benefit over wildtype marginals, which often failed to justify the added compute^21,75^. However, these studies did not consider whether different scoring strategies provide added value for model calibration, as we address next.

### Guiding calibration with class-conditional feature distribution shifts

To study the correlation between label and feature shifts, we must quantify conditional feature distribution shifts. For a given attribute M and its complement ¬M, we compute the conditional Jensen-Shannon divergence^76^ between the two groups’ feature entropies X (Methods):

$$\text{divergence}=\sum_{l\in \{\text{pathogenic},\text{benign}\}}JSD(P(X|M,l)\|P(X|\neg M,l))$$

**Figure 2.**
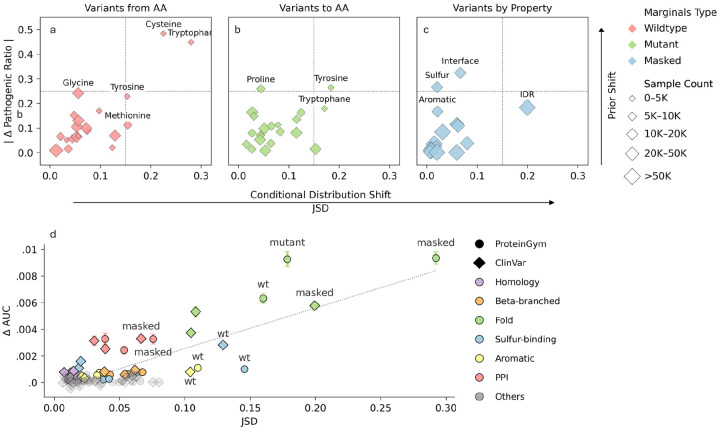
Conditional feature distribution and AUROC. **a-c**. Relationship between label shifts (y-axis; absolute deviation from the global pathogenic ratio) and class-conditional feature distribution shifts (x-axis; Jensen-Shannon divergence between subgroup partitions). Variants are grouped by substitutions of the wild-type amino acid (a), substitutions to specific amino acids (b), and residue property-based variants (c). Diamond size denotes the smaller subgroup in each partition, and color indicates the scoring strategy (pink - WT, green - MUT, blue - MSK). **d**. Change in global AUROC (y-axis; relative to the uncalibrated model) as a function of class-conditional shift (x-axis; Jensen-Shannon divergence). Pearson correlation r = 0.79, p < 2e-21. Calibration was performed on a balanced training set consisting of one-third of the data (Methods), leaving two-thirds for testing. Error bars indicate ±1 standard deviations across 100 non-parametric bootstrap test iterations per fold. Circles and diamonds correspond to results on the ProteinGym and ClinVar_HQ benchmarks, respectively. Symbol color indicates the calibrated residue attribute (legend); grey transparent symbols represent other tested attributes (Table S2).

Label shifts are quantified as the absolute difference between the pooled pathogenic ratio across the dataset and the ratio observed for the specific attribute (Table S1). We analyze three classes of variants: substitutions of the wildtype amino acid (AA) (Figure 2a), substitutions to specific AA (Figure 2b), and variants involving residues with specific properties (Figure 2c). Each class is evaluated using the feature-vector entropy computed from the input sequence most appropriate for capturing its signal: wildtype sequence for wild type substitutions, mutant for substitutions to target AAs, and masked for residue-property attributes, which yield the most significant divergence.

Pronounced label shifts can occur without corresponding feature distribution shifts, likely reflecting labeling biases or limited model capacity, while the reverse is rarely observed (Figure 2a–c). Because feature distribution shifts usually coincide with prior shifts, we hypothesized that subgroups with stronger feature shifts are optimal targets for calibration, reflecting true label differences captured by the model. Accordingly, we examined whether entropy divergence between an attribute and its complement predict calibration-related AUROC gains (Figure 2d, Table S2).

Simple calibration was performed by fitting separate linear logistic rescaling functions, per subgroup, on a balanced training set (Methods). We find a strong positive correlation between class-conditional feature distribution shifts and the calibration contribution to AUROC $\left(r=0.79,p<2e^{-21}\right)$. Since contributions to total AUROC can be affected by group sizes, such as the lower number of residues in PPIs compared to disordered regions, we also report a relative contribution metric, defined as the total gain in AUROC divided by the number of variants in the smaller attribute split (Figure S3).

The analysis highlights that residue disorder-level and interface status provide substantial AUROC gains in ESM1b when calibrated separately. Aromatic and sulfur-binding residues showed limited shifts under masked sequence entropy but stronger ones with wild-type sequence, and calibrating on the latter significantly improved AUROC.

### Benchmarking discrimination across 23 VEPs reveals profound residue-level variation

To generalize our findings beyond ESM1b, we benchmark calibration across 23 SOTA VEPs (Table 3) using a ClinVar-derived dataset curated by Radjasandirane et al.^9^ (ClinVar_BM, Methods) to minimize data leakage and circularity.

A key challenge when benchmarking multiple supervised and unsupervised VEPs is that scores vary substantially in range, classification thresholds, interpretability (e.g., probabilistic versus heuristic scores), and by models that report only calibrated outputs. To enable comparison on a common scale, we map all scores to [0,1] range via monotonic normalization or calibration (Methods), which preserves discriminative performance but leaves thresholds non-interpretable.

**Figure 3.**
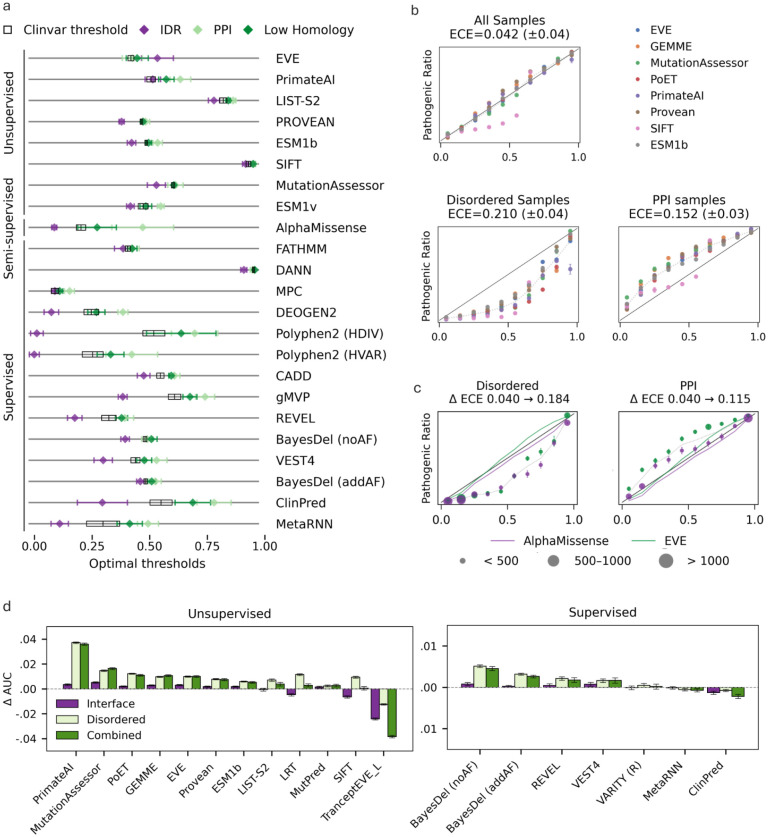
Residue-wise calibration. **a.** Shifts in optimal discrimination thresholds (diamonds; maximal J-statistic) across normalized model scores in the ClinVar_BM dataset. Error bars indicate ±1 SD over 1,000 non-parametric bootstrap iterations. Black rectangle marks the naive threshold (mean ± SD) across ClinVar_BM. **b.** Reliability histograms show predicted confidence (x-axis, 10 equal-width bins) versus observed pathogenic frequency (y-axis) for globally calibrated models using logistic regression. Perfect calibration follows y=x; dotted lines show the mean trend across models. Circles mark per-model bin frequencies; error bars indicate ±1 SD across 100 global calibration iterations. Panels display the full ProteinGym dataset (top left), disordered variants (top right), and PPI variants (bottom left). ECE = the mean weighted Expected Calibration Error across models. **c.** Miscalibration of EVE and AlphaMissense calibrated scores. Green and purple lines show naive calibration trends for EVE and AlphaMissense, respectively; circle size denotes bin sample count. Other details as in (b). **d.** Effect of residue-specific calibration on global AUROC for unsupervised (left) and supervised (right) VEPs. Y-axis shows ΔAUROC from baseline; error bars = ±1 SD over 1,000 bootstrap iterations.

To compare discrimination across variant properties, we examine raw model scores prior to any downstream calibration. Scores outside [0,1] are mapped to this range using modified min-max normalization and flipped when needed so that higher values indicate pathogenicity (Methods). Optimal pathogenicity thresholds are then derived globally and within each variant subgroup by maximizing the Youden J-statistic over 1,000 bootstrap iterations (Methods). We focus on variants in PPI and disordered regions, previously shown to exhibit systematic under- or over-estimation, as well as variants with few homologs, where homology-based models may be particularly sensitive (Figure 3a). We also provide an extended analysis (Figure S4, Table S3).

Classification thresholds substantially shift for variants in PPI and disordered regions across nearly all models, with many showing shifts in both subgroups. Variants in sulfur-containing residues (methionine, cysteine) also display consistent shifts. Among tested VEPs, FATHMM and SIFT were the most stable, showing no significant changes. Furthermore, shifts tend to be model-specific, for example, polar and aromatic residues show pronounced shifts in AlphaMissense and gMVP, less evident in other models, likely reflecting the effect of structural features. These shifts do not necessarily track overall performance (Figure S5, Table S3) but must be corrected to ensure consistent behavior across variant subgroups.

Despite these findings, the standard practice when using VEPs is to apply a single global threshold. Some methods allow more stringent cutoffs to tune precision, but to the best of our knowledge, none of the evaluated models recommend thresholds specific to different variant types. Consequently, global thresholds provide no guarantees of performance within individual subgroups, an issue recently addressed by Fawzy et al. for variants in IDRs^58^. By contrast, threshold variation is minimal at the protein level, Frazer et al. (2021) reported that the EVE evolutionary index shows a nearly uniform classification boundary across proteins. These observations underscore the need for residue-level rather than protein-level calibration to capture subgroup-specific variation in model behavior.

### Globally calibrated VEPs are not robust at the residue level

Multicalibrated models aim to provide reliable probabilities across diverse subpopulations. While some VEPs output probabilities inherently (SIFT, PolyPhen), others incorporate downstream calibration. EVE combines global and per-protein GMM calibration while AlphaMissense applies a global logistic regression fitted on a subset of ClinVar variants. To demonstrate the effect of global calibration we naively calibrate the reported raw scores of eight SOTA unsupervised VEPs using the ProteinGym Clinical Substitution benchmark. These models span sequence-based, alignment-based, and clinically derived predictors. Global calibration was performed using a logistic regression trained on 100 balanced random subsets of 6,000 variants (~10% of the dataset; Methods). To assess calibration quality, we used reliability histograms^77,78^, which plot model accuracy as a function of predicted confidence (Figure 3b, Methods). For a perfectly calibrated model, the curve follows the identity line. Deviations above or below this line indicate overconfidence and underconfidence in predictions, respectively. To quantify miscalibration, we measured the weighted average Expected Calibration Error (ECE)^79^ (Methods), where ECE = 0 indicates perfect calibration and larger values reflect increasing miscalibration.

Following global calibration, all models achieve near-perfect calibration, with an average ECE of 0.042 (Figure 3b, top left). However, when restricted to variants in disordered regions or in PPIs, calibration consistently breaks down across all models (Figure 3b, bottom), showing underconfidence in disordered residues and overconfidence in PPIs. In these subgroups, ECE increases up to fivefold relative to the globally calibrated baseline. Miscalibration also worsens at lower prediction confidence, where accuracy deviates further from the identity line.

We next examine the pre-calibrated scores provided by EVE and AlphaMissense (Figure 3c), using a subset of ClinVar variants not included in the AlphaMissense calibration (ClinVar_AFM, Table 2, Methods). The same patterns persist, with many variants receiving confidence estimates that deviate substantially from the global calibration baseline. Notably, AlphaMissense provides relatively well-calibrated scores for variants in PPIs, which are likely better captured when structural features are incorporated.

### Residue-level calibration often improves global AUROC across VEPs

As shown above, miscalibration is pervasive across residue-level subgroups, underscoring the need for multicalibration in VEPs. Because reliable probabilities alone are insufficient, we also examine how multicalibration affects discriminative performance by evaluating overall and per-protein AUROC (Methods) on the ProteinGym Clinical Substitution benchmark. Calibration follows the ESM1b protocol, fitting separate logistic regression models per subgroup (Methods). We test three schemes: (1) interface vs. non-interface, (2) disordered vs. ordered, and (3) a joint scheme further splitting ordered residues (disordered residues were not subdivided due to limited data). Although these subgroups are miscalibrated across models, calibration ideally depends on model-specific features, requiring raw model scores which are often unavailable.

Residue-specific calibration not only significantly improves calibration within these subgroups (Figure S6) but also improves overall AUROC across nearly all unsupervised models, with modest gains even for supervised predictors, and without reducing per-protein AUROC (Figure 3d). Improvements appear with as few as 250 training samples (Figure S7). The only exception is Trancept_EVE_L, where AUROC decreases. Because overall AUROC is dominated by cross-protein discrimination, these gains are attenuated when evaluating per-protein AUROC. Together, these results reinforce that VEP scores should be interpreted and calibrated in a context-dependent manner across variant subgroups.

### RaCoon - Residue-aware Calibration of conditional distributions

Leveraging our finding that residue-level calibration, guided by feature distributions, improves both reliability and accuracy, we design a calibrated VEP that guarantees consistent performance across variant subgroups. In addition, we aim to develop a clinically interpretable model capable of producing meaningful pathogenicity probabilities. We chose ESM1b as the base model for RaCoon due to its strong performance, unsupervised nature, and lack of reliance on MSAs^4,8,9^.

**Figure 4.**
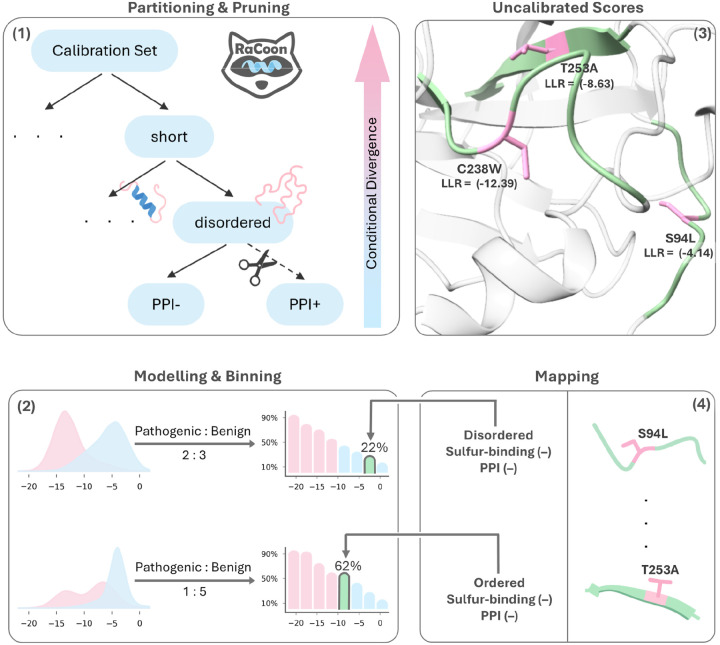
The RaCoon pipeline. **(1)** a calibration set is partitioned across binary residue attributes, with attributes showing larger conditional feature distribution shifts placed closer to the root. Leaves with insufficient data are pruned (scissors icon). **(2)** Nodes’ pathogenic and benign LLR score distributions are modeled using two GMMs and the pathogenic to benign ratio is estimated. Synthetic samples are subsequently drawn from the GMMs according to the estimated ratio to construct calibration histograms (x-axis LLR scores, y-axis pathogenic fraction). **(3)** Uncalibrated per-variant LLR scores (pink sticks, PDB 2pcx) are obtained from ESM1b. **(4)** Each variant is mapped to its node and histogram bin to yield a residue-wise calibrated pathogenicity probability.

Our calibration approach (Figure 4) targets three binary residue attributes identified earlier as optimal calibration candidates: disorder level, interface status, and sulfur-binding (methionine or cysteine versus other residues). Because inference on long proteins in ESM1b (>1,022 residues) requires a sliding-window approach (Methods), which has been shown to affect performance^4,80,81^, we treat protein length as a separate calibration subgroup. Accordingly, we introduce an additional technical division between residues in long and short proteins.

The RaCoon pipeline consists of four main steps (Methods). First, attribute combinations are partitioned into a calibration tree (Figure S8), where each node represents a unique set of binary residue attributes, nodes with insufficient data are subsequently pruned. Second, per-node feature distribution is estimated by fitting two GMMs, over a small subset of pathogenic and benign variants’ LLR scores. Third, LLR scores are binned into pathogenicity histograms by sampling from the GMMs according to the estimated node’s label ratio. Each bin reflects the proportion of pathogenic samples for a given LLR range. Finally, at inference time, variants are mapped to their corresponding node and histogram bin, mapping LLR scores to interpretable calibrated probability scores.

Importantly, the calibration pipeline is never directly exposed to specific labeled examples. Both the base model and the downstream calibration hinge solely on modeled benign and pathogenic score distributions and their priors. By modeling these distributions with GMMs, the calibration requires minimal labeled data and, as we later show, avoids overfitting and data leakage. Although estimating label ratios remains susceptible to annotation biases, it holds important biological signals that we aim to model. As previously addressed, restricting calibration to attributes with strong feature distribution shifts and pruning nodes with insufficient data increases the likelihood that estimated ratios reflect genuine biology rather than noise. RaCoon’s consistent performance across datasets indicates that it calibrates to global score distributions rather than dataset-specific biases.

### RaCoon outperforms ESM1b on clinical and experimental datasets while improving calibration

**Figure 5.**
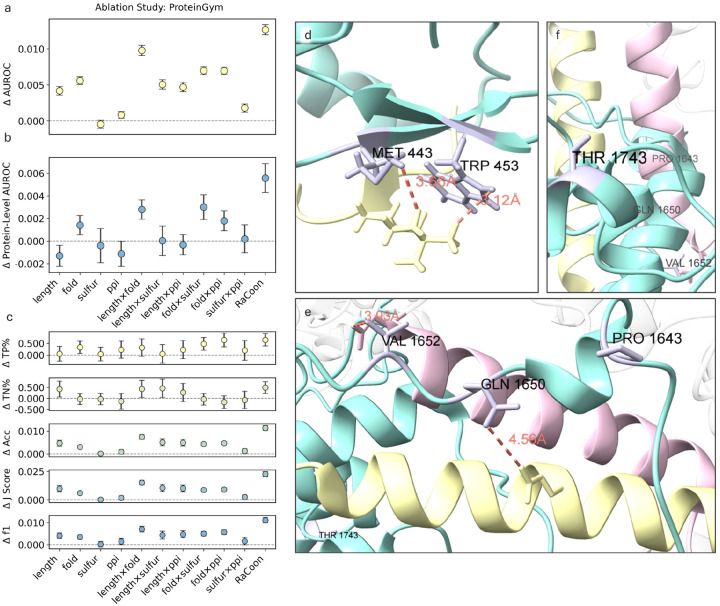
Performance evaluation ClinVar_HQ. **a-c.** Changes in overall performance relative to uncalibrated ESM1b. Error bars denote ±1 SD across 100 randomized training iterations, each evaluated with 100 non-parametric bootstrap test resamples (a) overall AUROC (b) per-protein AUROC (c), and discrimination metrics. Per-protein AUROC was computed for sequences containing ≥10 variants with at least one pathogenic and one benign label. TP = true positive; TN = true negative; thresholds were optimized per model to maximize the Youden J-statistic. **d-f.** Structural examples of variants correctly reclassified after calibration in RAG2 (PDB 8T4R, d) and ARID1A (PDB 6LTJ, e-f). ClinVar-annotated variants (purple sticks) are shown with interface distances (orange, Å). Interface partners: yellow - Histone H3 (RAG2) and SMCE1 (ARID1A); pink - SMRD1.

To evaluate the performance of RaCoon against the baseline ESM1b, we measure AUROC and per-protein AUROC (Methods). For clinical data, we use the ClinVar_HQ dataset, for experimental data, we use the ProteinGym Clinical Substitution benchmark (Table 2, Figure 5a–b, Figure S10). All reported results are obtained using 100 random training (calibration) iterations, each tested using 100 independent non-parametric bootstrap test iterations. Classification thresholds used are calculated by maximizing the Youden J-statistic over the entire test set.

On ClinVar_HQ, RaCoon achieves an AUROC of 0.941 ± 0.001 compared to 0.930 ± 0.000 using raw LLR scores from ESM1b. A similar improvement is observed on ProteinGym, where RaCoon reaches an AUROC of 0.924 ± 0.001 compared to 0.912 ± 0.000 of the naive model. For per-protein AUROC, the calibrated model provides a moderate but statistically significant improvement on ClinVar_HQ (0.904 ± 0.001 vs. 0.899 ± 0.001), and on ProteinGym (0.909 ± 0.002 vs. 0.903 ± 0.001). Moreover, the ROC curve of RaCoon consistently lies above that of the naive model, indicating superior performance across all classification thresholds (Figure S11).

To ensure performance gains are not stemmed from overfitting of dataset-specific distribution and prior estimations, we evaluate RaCoon’s performance on ProteinGym using ClinVar as the calibration set (Figure S10d–f). Racoon delivers similar results, improving AUROC to 0.921 ± 0.001 compared to the uncalibrated model (0.911 ± 0.00), indicating that the calibration procedure generalizes beyond a single dataset.

A key aspect of RaCoon is its ability to provide multicalibrated predictions across variant subgroups. To evaluate calibration quality directly, we report both ECE and the more stringent Maximal Calibration Error (MCE) for every calibrated node (Methods). RaCoon achieves consistently low calibration error across all subgroups within and across datasets (Table S4). On ClinVar_HQ, 15 of 16 calibrated nodes obtain ECE values < 0.1, with only a single node, corresponding to a small subgroup, reaching 0.26. All but three nodes achieve MCE scores below 0.2. Moreover, RaCoon maintains calibration across datasets. When calibrated on ClinVar and tested on ProteinGym, all nodes maintain ECE < 0.13, and all but two nodes maintain MCE < 0.2. In comparison, naive calibration using logistic regression over ClinVar_HQ results in 8 out of 16 nodes receiving an MCE > 0.3.

### Ablation studies identify specific contributors to performance gains

To disentangle the contributions of individual components in our calibration pipeline, we compare naive ESM1b to our calibrated model under multiple single and pairwise feature ablations (Figure 5a–b, Figure S10). In addition to AUROC, we report complementary binary classification metrics, all averaged over repeated training and bootstrap iterations, and reported as changes relative to the uncalibrated ESM1b on variants not seen during the calibration process.

Calibration to protein length and fold emerge as key contributors to AUROC across both datasets, with largely additive effects. Notably, differences in protein length that are not reflected in the class-conditional feature distribution, remain important, underscoring the need to correct for context length in transformer-based models. Pairwise calibration to fold and sulfur-binding residues also yielded consistent gains.

While several attributes contributed to AUROC improvement, their impact at the optimal classification threshold are more subtle (Figure 5c), illustrating the limitations of global thresholds. AUROC-based gains primarily reflect improved global ranking^82,83^, whereas per-protein AUROC shows smaller gains. Per-protein AUROC is a less stable metric for proteins with a few labeled variants^84,85^ and does not account for inter-protein ordering, imperative for genome-wide screening^4^.

### RaCoon improves borderline variant interpretation in RAG2 and ARID1A

Complete calibration yields additional AUROC gains and significantly improves threshold-based metrics, enhancing discrimination of both benign and pathogenic variants. These benefits are most apparent for borderline cases, where misclassification risk is highest, as illustrated by example cases in RAG2 and ARID1A (Figure 5d–f).

RAG2 is a core V(D)J recombinase component whose mutations cause severe immunodeficiencies^86–88^. Two variants at residue M443 (M443I pathogenic, M443T likely-pathogenic^89,90^), are misclassified as benign by ESM1b raw LLR scores. RaCoon correctly identifies them as pathogenic, assigning interpretable and calibrated probabilities of 73% (M443I) and 74% (M443T). This improvement reflects methionine’s classification as a sulfur-binding residue, which places these variants into a distinct calibration subgroup. In contrast, a nearby W453 variant (non-sulfur binding) affecting the same interaction is correctly classified as pathogenic by both models.

Context dependence is illustrated by the LLR scores mappings: M443I (−7.03) maps to 73% pathogenic probability, whereas W453R, assigned to a different node, receives a more negative LLR (−9.25) yet a similar calibrated probability (74%). Although more negative LLRs typically imply stronger pathogenicity, this example shows how RaCoon adjusts predictions according to local distributional context rather than LLR magnitude alone.

An opposite effect is seen in ARID1A, where calibration correctly reclassifies 12 variants from pathogenic to benign. ARID1A is a core subunit of the BAF (SWI/SNF) chromatin-remodeling complex^91^ and a major tumor suppressor recurrently altered across cancers^92–95^. Its large size, long disordered regions, and multiple PPIs make variant interpretation particularly challenging.

Two benign ClinVar variants, P1643T and T1743M (Figure 5e–f), receive uncalibrated LLRs in the pathogenic range (−8.91 and −8.50) but are correctly reclassified as benign once residue context is incorporated. Notably, P1643T lies near the SMARCD1/SMARCE1 interface, yet RaCoon maps it to a pathogenicity ratio of 29%. Nearby variants (Q1650H, V1652A) remain misclassified, likely due to predicted interface involvement, but their calibrated probabilities (46% and 44%) are borderline. For comparison, an interface-associated variant in KCNQ2 (Q341H), with a comparable LLR (−9.05), receives a much higher calibrated pathogenicity of 68%.

Together, these examples illustrate how region-specific score mappings can yield different pathogenicity estimates for similar LLRs, highlighting the importance of local residue context.

### GMM-based sampling provides efficient calibration with minimal labeled data

We next quantify the contribution of GMM-based sampling relative to naive direct binning (Figure S9a–b, Methods). Two results stand out. First, GMM-only calibration with as few as 100 training samples performs similarly to direct binning, which requires tens of thousands of labeled variants. This likely reflects the GMMs’ denoising effect achieved through their constrained expressiveness, which likely improves sampling efficiency. Second, although GMM performance improves roughly linearly with training size, the number of retained calibration subgroups decreases due to pruning (Figure S9b). To balance accuracy with subgroup coverage, we set the final training size to 400 samples, which preserves most calibration nodes.

Finally, we assess the robustness of our calibration method to potential data leakage. To ensure that neither the base model nor the calibration step benefits from memorizing specific examples, we compare performance when models are evaluated on their own training data versus on held-out test data with matched pathogenic-benign ratios (Methods). Even under this extreme setting, performance remains unchanged on both ClinVar_HQ and ProteinGym. Although our pipeline already separates GMM training from test samples, this ablation confirms that data leakage is not a practical concern for our framework.

## Discussion

Recent years have seen a significant progress in missense variant effect prediction, leading to growing adoption in both research and clinical variant classification^29,47^. As VEPs become more deeply integrated into clinical workflows, the need for reliable interpretation frameworks that account for biases in training and evaluation has grown urgent. To develop effective calibration protocols, we must address the question: what should we calibrate for?

A common strategy is to sidestep this question by performing global calibration across the entire dataset or by calibrating on a per-protein basis^6,29,31^. These approaches implicitly treat the attributes driving miscalibration as latent variables, aimed to be captured by global protocols, without explicitly identifying their underlying sources. In contrast, our study takes a targeted approach, leveraging the distribution of model-predicted probabilities to detect variant subgroups that require distinct calibration.

We set out to explore the naive approach, systematically searching for label shifts across variant subgroups using clinical annotations. Our findings align with and extend previous works, detecting pronounced label shifts in several residue categories^42,57–59^, including intrinsically disordered, polar, aromatic, and sulfur-containing residues (Figure 1a, 2a). Yet it remains unclear whether these shifts reflect genuine biological mechanisms or arise from labeling biases. Our first key observation is that unsupervised models, unaffected by labeling biases, may disentangle these effects. As SOTA models better capture biological constraints, label shifts driven by biological mechanisms are expected to manifest as changes in their class-conditional feature distributions (Figure 1c,d). We indeed move to show that divergence in feature distribution is a strong predictor of label shifts (Figure 2a). This insight may also help correct for labelling biases in supervised model training on biased datasets.

Calibrating by residue-level attributes can be viewed as introducing additional context features at inference time. Accordingly, for an attribute to be suitable for calibration, it must: (1) diverge from the average training distribution^66,96^, and (2) this divergence must be detectable by the model^51,55^. Together, these criteria make class-conditional feature distribution shifts particularly informative for guiding calibration. While calibration is independently important for VEPs, our second key insight is that shifts in class-conditional feature distributions also strongly predict AUROC gains following calibration (Figure 2b). Although our analysis focuses on ESM1b, the attributes identified (specifically fold and PPI) improve AUROC across nearly all models tested (Figure 3d), underscoring the general applicability of this finding. Notably, conditional probabilities vary between models, hence, calibration should ideally be performed on a per-model basis.

To demonstrate the importance of residue-level calibration, we examine both the discriminative power and interpretability of VEP scores across dozens of residue attributes. We and others^58^ report widespread shifts in optimal classification thresholds and score reliability (Figure 3a, Figure S5), which vary across models and tend to be model-specific. Together, these findings emphasize that VEP predictions must be interpreted in a context-dependent manner and reinforce the need for calibration strategies tailored to distinct variant subgroups.

Current VEP benchmarking practices typically assess performance globally^7,9^ or per-protein^8^ without explicit consideration of residue-level variation. Such evaluations may overlook subgroup-specific reliability. As our results suggest this can lead to variability in calibration across variant types (Figure 3a–c), highlighting the potential value of incorporating residue-level assessments into benchmarking protocols.

Building on these insights, we introduce RaCoon (Figure 4), a multicalibrated variant effect predictor based on ESM1b^4,97^ that applies residue-wise calibration across key attributes identified in this study. The framework learns class-conditional LLR distributions using GMMs and converts them into interpretable pathogenicity probabilities through calibration histograms. It requires minimal supervision and inherently limits overfitting and data leakage. Importantly, the approach can be readily extended to other unsupervised VEPs, enabling calibration guided by model-specific conditional feature distributions. RaCoon substantially improves the AUROC compared to the uncalibrated model but more importantly, produces context-aware, interpretable predictions at test time (Figure 5). The model and calibration framework are publicly available via an open-access server.

## Online Methods

### Datasets Curation (Table 2)

#### ClinVar_HQ.

The complete ClinVar^7^ data was downloaded in GRCh38 VCF format (July 2025). Records missing core ClinVar annotations (CLNSIG, CLNVC, CLNREVSTAT, or CLNHGVS) were excluded. We retained only single-nucleotide variants and required a review status ≥1 star, based on the CLNREVSTAT stars mapping. This filtering step removed conflicting variants, variants lacking clinical classification, and those with no assertion criteria, yielding 1,751,722 variants. Next, all transcript identifiers were resolved using the Entrez API^98^, and only variants with valid protein-level variant symbols were retained (that is, those beginning and ending with canonical amino acids whose wild-type residue matched the reference transcript). Variants of uncertain significance were excluded, retaining only variants annotated as Likely pathogenic, Pathogenic/Likely pathogenic, Pathogenic, Benign, Benign/Likely benign, or Likely benign. These categories were mapped to binary labels. Finally, we unified duplicate variants, sharing the same binary label in the same transcripts and removed variants with contradicting labels. The resulting ClinVar_HQ dataset contained 171,196 variants across 14,564 unique protein sequences, of which 52,634 were pathogenic and 118,562 benign.

#### Well-annotated ClinVar proteins.

This dataset was compiled directly from the ClinVar_HQ dataset, retaining only sequences with at least ten variants, of which we required a minimum of four pathogenic and four benign variants. The final dataset consists of 42,056 pathogenic and 44,155 benign variants across 1,285 protein sequences.

#### ClinVar_BM.

This dataset represents a rigorously filtered subset of ClinVar designed for benchmarking VEPs and minimizing circularity and annotation biases. We use the dataset recently published by Radjasandirane *et al*.^9^, which includes 14,054 variants (11,757 pathogenic and 2,296 benign), all published after May 1, 2021. Variants that also appeared in the Humsavar dataset^99^ or showed unbalanced annotations (ratio exceeding 60/40) were excluded. All remaining variants were verified to represent valid protein-level alterations. Because transcript identifiers were not provided, variants were mapped to sequences using gene symbols, leveraging the transcript mappings established for the ClinVar_HQ dataset.

#### ClinVar_AFM.

AlphaMissense, published in 2023, includes a supervised calibration process that could potentially introduce variants present in the ClinVar_BM dataset. To prevent such circularity, we used a subset of ClinVar variants published by Cheng et al.^6^ that were explicitly excluded from the AlphaMissense calibration process. We removed variants for which no matching transcript could be found in the ClinVar_HQ dataset, ensuring that all remaining variants met our established filtration criteria. The final dataset consists of 56,368 variants, including 18,765 pathogenic and 37,603 benign variants across 6,272 unique protein sequences.

#### ProteinGym.

Throughout the study we use the ProteinGym zero-shot clinical substitution benchmark^8^ obtained in June 2025. For attribute analysis we use the provided protein sequences, labels are assigned using the *DMS_bin_score* column. The benchmark contains 62,727 variants (32,000 pathogenic and 30,727 benign) across 2,525 unique protein sequences.

#### VEPs used for Benchmarking (Table 3).

Model scores were computed using Ensembl VEP^100^, except for EVE and AlphaMissense that were queried directly from the published precomputed datasets.

### Classification Performance Metrics

#### Global and per-protein AUROC.

The global and per-protein Area Under the Receiver Operating Characteristic curve (AUROC) are widely used metrics to assess VEP performance. For all models, we assume that higher scores indicate greater pathogenicity. This assumption holds for all cases where calibration using logistic regression was performed (Figure 2b, Figure 3d). When raw scores are analyzed (Figure 3a), we first learn a monotonic rescaling function using the modified min-max normalization described below and, if necessary, flip the scores so that values closer to 1 correspond to pathogenic variants and those closer to 0 to benign variants. Because this transformation is strictly monotonic, it preserves the global rank order of scores and thus does not affect the AUROC.

The per-protein AUROC was computed by first calculating the AUROC independently for each protein sequence and then averaging across all valid sequences. Proteins lacking either pathogenic or benign labels were excluded from the mean calculation. Consistent with the ProteinGym benchmark protocol, we report the unweighted mean of per-protein AUROCs. We also tested a weighted-average variant, in which each protein’s contribution was proportional to its number of annotated variants; however, the results were nearly identical to the unweighted mean, and thus only the standard approach is reported.

All AUROC computations were performed on held-out test sets, strictly excluding any samples used during the calibration process. When comparing RaCoon to the uncalibrated ESM1b model, we always evaluated performance on identical test sets to ensure a fair comparison. We note that RaCoon’s ablation experiments (Figure S9) involved multiple calibration settings, which altered the composition of the resulting test sets leading to slight variations in reported AUROC values across experiments.

#### Optimal classification thresholds.

Optimal classification thresholds were determined by maximizing the Youden J-statistic^116^ across all possible decision thresholds. That is, we chose the threshold that maximized the difference between the true positive rate and the false positive rate:

(1) $$c^{*}=\text{arg}\;{\text{max}}_{c}\{TPR(c)-FPR(c)\}$$

J-score is often preferred over accuracy in imbalanced test sets and, unlike the F1-score, it also accounts for true-negatives^117^. These make it particularly suitable for clinical and diagnostic tasks^118^ and it has also been adopted for VEP benchmarking^58^.

For the uncalibrated ESM1b model, the optimal threshold determined by maximizing the J-score on the ClinVar_HQ dataset was −8.22, which differs from the paper suggested threshold of −7.5. While the original rationale for the −7.5 threshold is not specified, using the J-score–derived threshold resulted in higher classification accuracy in our evaluation.

### Residue Attributes

All attributes were directly inferred from the protein sequence associated with the variant. Predictors used as well as associated thresholds are specified in Table 4.

#### Disordered regions.

Residues in disordered regions were inferred using ALBATROSS Metapredict v1.3^44,119^. We precomputed disorder scores per residue for all isoforms of reviewed human proteins in UniProt^120^, as well as for all transcripts in our datasets. Each residue was locally mapped to the corresponding disorder score based on its position within the matching transcript. Residues with scores above 0.7 were defined as disordered. Although the suggested threshold is 0.5, we found that a 0.7 cutoff better aligned with previous disorder estimates^44,45^, predicting approximately 28% of residues as disordered compared to 32% using the 0.5 threshold. Increasing the threshold mainly changes the predictions of residues in transition regions therefore, aiming for more rigorous disorder prediction, we use the 0.7 threshold.

#### Protein-protein interface.

To predict residues involved in protein–protein interfaces (PPIs), we used PIONEER^73^. We obtain high-confidence interface predictions for all reviewed human UniProt sequences. PIONEER integrates binary predictions from three sources: the PIONEER model, experimentally resolved complexes from the Protein Data Bank (PDB)^121^, and homology models. A residue predicted to be involved in a PPI by any of these sources was considered PPI-positive. Predictions were available for 74% of variants in the ClinVar_HQ dataset, of which 9% were predicted to participate in PPIs (N = 11,558). In the ProteinGym dataset, predictions were obtained for 77% of variants, with 12% predicted to participate in PPIs (N = 5,749).

#### Homology.

To distinguish between sequences with few versus many homologs, we directly queried the UniRef sequence clusters^122,123^. A key advantage of estimating homology using UniRef clusters is that it avoids systematic similarity searches across the entire human proteome, which would be impractical at inference time. We experimented with both UniRef90 and UniRef50 clusters, testing multiple thresholds to maximize separation of class-conditional distributions. We ultimately used UniRef90, defining sequences with ten or fewer homologs as low-homology. Using this threshold, approximately 9% and 7% of sequences in the ClinVar_HQ and ProteinGym datasets, respectively, were categorized as low-homology. While lower thresholds increased the class-conditional separation, they also produced very small subgroups. A threshold of nine therefore provided a suitable balance between subgroup size and distributional contrast.

#### Physico-chemical properties.

We compiled an extensive set of amino acid physicochemical properties previously reported to influence the functional impact of missense variants (Table 4)^59,60,70,124,125^.

### Log Likelihood Ratio Scores (LLR)

Throughout this study, we use LLR score derived from ESM1b as a quantitative measure of predicted pathogenicity. Given a protein sequence S with a missense variant at residue position i, the LLR score can be computed using either the wild-type (wt), mutant (mut), or masked (msk) sequence. These three inputs differ only at residue i, where the masked marginal substitutes the original residue with a special mask token.

Let $X\left({S^{t}}_{i}\right)\in R^{N_{AA}}$ denote the model output vector (logits) for position i in sequence type t∈{wt,mut,msk}, where $N_{AA}$ is the number of amino acid tokens.

We define the LLR score as:

(2) $$LLR^{\left(t\right)}=\text{log}\left(\text{softmax}\left(X{\left({S^{\left(t\right)}}_{i}-\left(X{\left({S^{\left(t\right)}}_{i}\right)}_{wt-AA}\right)\right)}_{\text{mut}-AA}\right.\right.$$

That is, we first subtract the wildtype entry from the entire output vector of residue i, apply a softmax to the resulting log-odds ratios, and finally take the mutant entry of this normalized vector as the LLR score.

We note that in some cases, prior works computed the LLR scores by first applying the *log_softmax* function and then subtracting the wildtype score. However, this approach mathematically cancels the softmax normalization, reducing the computation to the raw logit difference. This follows directly from the definition of the softmax function. Given an unnormalized logits vector l it holds that:

$$\text{log}(\text{softmax}(l){)}_{\text{mut}-AA}-\text{log}(\text{softmax}(l){)}_{wt-AA}=\left(l_{\text{mut}-AA}-\text{log}\left(\sum_{j}e^{l_{j}}\right)\right)-\left(l_{wt-AA}-\text{log}\left(\sum_{j}e^{l_{j}}\right)\right)\;=l_{\text{mut}-AA}-l_{wt-AA}$$

Although we did not observe substantial performance differences using this formulation, all reported comparisons in this study were performed using the implementation described in (2).

#### Extending LLR to long sequences.

ESM1b has a maximum input context of 1,024 tokens, which limits the model’s ability to process full-length proteins exceeding this length. To handle such cases, we applied a sliding-window approach with a window size of 1,022 residues and a 250-residue overlap between consecutive windows. For each window, the model computes ESM1b scores for the central 1,022 − 2×250 positions, excluding the overlapping regions to avoid redundancy. In the first and last windows, the terminal residues are also included to ensure full sequence coverage. This strategy preserves the model’s maximal input length while preventing indexing errors and maintaining smooth coverage across long proteins. Following window extraction, LLR computation proceeds identically to the method described above, using the wildtype, mutant, or masked representations as appropriate.

#### Mutual Information (MI) Estimation.

To quantify the association between residue attributes and pathogenicity (Figure 1b), we computed the MI between selected attributes and the binary clinical label (Table 5). At the residue level, MI was calculated directly from the binary attribute indicator and the pathogenic labels using scikit-learn’s *mutual_info_score*. At the protein level, each proteins’ residues were assigned to one of three groups, based on the proportion of residues exhibiting the attribute (corresponding to the violin bins in figure 1b), MI was computed between these group assignments and the pathogenic labels of the residues. For both analyses, uncertainty was estimated using 1,000 non-parametric bootstrap iterations.

### Learning Gaussian Mixture Models Over LLR Scores

Throughout the study we use Gaussian Mixture Models (GMMs)^69^ to estimate the pathogenic and benign LLR distribution. We follow a similar scheme of extreme outliers removal, sampling, and fitting for all GMMs trained.

#### Extreme outliers removal (modified z-score).

For all experiments using ESM1b, including RaCoon, we removed extreme outliers from the full dataset (without separating pathogenic and benign variants) using the modified z-score^126,127^. For each variable, we first computed the median and the Median Absolute Deviation (MAD). Let $\overset{ˆ}{x}$ denote the median LLR score, we define:

(3) $$\text{MAD}=\text{median}\left(\left|x_{i}-\overset{ˆ}{x}\right|\right)$$

The modified z-score for each observation is then defined as:

(4) $$z_{i}=0.6745\times \frac{x_{i}-\overset{ˆ}{x}}{\text{MAD}}$$

Samples with $\left|z_{i}\right|>4.25$ were considered extreme outliers and excluded from GMM training. This approach removes outliers symmetrically from both tails of the distribution, effectively eliminating extreme benign and pathogenic scores. The chosen threshold corresponds to a highly stringent cutoff intended to exclude only severely irregular samples that could otherwise distort GMM fitting. For instance, across the full CalinVar_HQ dataset, this process excluded 576 variants corresponding to 0.33% of all entries.

#### Sampling.

We randomly sampled n LLR scores (after outlier removal) from the benign and pathogenic distributions. The exact number of samples varied across experiments and is explicitly reported in the Results section. For RaCoon, both GMMs were trained using 400 samples per class.

#### Fitting GMMs.

We fit separate GMMs for the benign and pathogenic samples. GMMs were implemented using the GaussianMixture module from scikit-learn, configured with two components and diagonal covariance (equivalent to full covariance in one-dimensional data), while all other parameters were kept at default values.

The number of components was chosen by computing the Bayesian Information Criterion (BIC)^128^ across models with increasing component counts and selecting the point at which the BIC curve plateaued. This procedure minimizes the risk of overfitting while maintaining sufficient flexibility to capture multimodal score distributions.

### Calibration with Logistic Regression

We used logistic regression to learn separate calibration functions for distinct residue-level attributes. All experiments followed a consistent sampling and fitting scheme, as outlined below.

#### Sampling.

In highly imbalanced datasets such as ours, standard maximum-likelihood estimation tends to be dominated by the majority class, shifting the fitted decision boundary toward the prevalent label and degrading the model’s ability to correctly rank samples across classes^129^. Training logistic regression on balanced subsets has been shown to improve overall ranking quality, enhance classifier discrimination, and mitigate majority-class bias^130,131^.

To fit each logistic regression model, we randomly sampled balanced datasets of pathogenic and benign variants. For Figure 2, the full dataset was partitioned into three disjoint subsets. In each iteration, one-third of the data was used for training, while the remaining two-thirds served as the test set. Within each training subset, we sampled equal numbers of pathogenic and benign variants by downsampling the majority class. This ensured balanced training across all residue attributes. Unused samples were returned to the test set. For Figure 3, we sampled 2n random examples, n pathogenic and n benign variants, per calibration task. The specific values of n varied across experiments and are explicitly reported in the Results section. For the MCE,ECE comparisons of RaCoon against a naively-globally calibrated ESM1b we sampled 6,000 random samples, 3,000 pathogenic and 3,000 benign variants.

#### Extreme outlier removal (percentile).

As per the GMM training protocol, we removed extreme outliers before fitting the logistic regression models. Specifically, samples falling in the top and bottom 0.1 percentiles (i.e., the most extreme 0.2% of values) were excluded. This proportion closely matches the number of outliers removed using the modified z-score method. However, because logistic regressions were fitted across multiple models with differing score ranges, we opted for a percentile-based criterion, which requires no model-specific adjustments (such as modifying the z-score threshold), ensuring consistent preprocessing across predictors.

#### Fitting logistic regression.

Following outlier removal logistic regression (LR) models were fitted using the scikit-learn *LogisticRegression*. The model was trained with the Limited-memory Broyden–Fletcher–Goldfarb–Shannon (L-BFGS) solver^132^, using otherwise default parameters.

Given x the raw LLR score and $c_{1},c_{2}$ the learnt scale and intercept coefficient of the fitted LR the calibrated score is defined as:

(5) $${\text{LLR}}_{\text{calibrated}}=\text{Sigmoid}\left(c_{1}•x+c_{2}\right)$$

### Data Normalization

To analyze raw VEP scores within a unified reference frame, we applied a modified version of the standard min-max normalization. First, extreme outliers, defined as values in the top and bottom 0.1 percentiles, were removed to prevent range distortion caused by outlier domination. Min-max normalization was then computed over the remaining inlier set. Following normalization, we verified that the mean score of pathogenic variants exceeded that of benign variants, if not, the scores were flipped by subtracting them from 1. Finally, the excluded outliers were reinstated, assigning a score of 1 to pathogenic and 0 to benign variants. Scores that were already bounded within the [0, 1] range were not renormalized but were flipped if necessary.

## Jensen-Shannon distributions divergence

To quantify divergence between class-conditional score distributions, we used the Jensen-Shannon divergence (JSD)^76^. JSD was empirically estimated by discretizing each subgroup’s normalized-entropy probability density (of the wild-type sequence) into histograms with 100 equal-width bins sharing ranging between 0–1.

The normalized entropy over a prediction vector x of size N is defined as:

(6) $$\text{NormalizedEntropy}(x)=1-\frac{H(x)}{\text{log}\;N}$$

To prevent zero-probability bins, a small smoothing factor $\varepsilon =e^{-12}$ was added to all bins. JSD was then computed using the SciPy Jensen-Shannon implementation, the divergence is then obtained by squaring the Jenson-Shanon distance. The reported JSD value is calculated as the sum of the independently estimated JSDs between benign variants and between pathogenic variants across the two subgroups.

To ensure JSD values are not affected by bin size, we computed the JSD for all attributes in and datasets analyzed in figure 2d using four bin resolutions (200, 100, 50, and 25 bins). Rank stability across bin sizes was assessed using Kendall’s τ, computed using the *SciPy kendalltau* implementation (Table 6). All pairwise comparisons showed strong positive rank correlations (τ≈0.7-0.92), indicating that the relative ordering of attributes is highly robust to the choice of bin size.

### Calibration Matrices

#### Calibration histograms.

Calibration, or reliability histograms^77,78^, were used in Figure 3 to visualize the reliability of the predicted model probabilities. Reliability histograms can only be applied to models that output probabilistic scores within the [0, 1] range. Histograms were constructed by discretizing models predicted confidence (probability) into 10 equally-spaced bins. We then compute the per bin empirical pathogenic frequency. For a perfectly calibrated model, the average predicted confidence should match the observed frequency (up to binning artifacts), yielding points close to the identity line. Deviations above or below this line indicate overconfidence or underconfidence, respectively.

#### Expected Calibration Error.

While reliability histograms provide intuitive visual insights, they are difficult to quantify and may be misleading when sample counts vary substantially across bins. To provide a quantitative measure of calibration, we computed the weighted Expected Calibration Error (ECE)^39^. For M bins $b_{1},\ldots ,b_{M}$ let $n_{b},\;{\text{freq}}_{b}$ and ${\text{conf}}_{b}$ denote, the empirical pathogenic frequency, and the mean predicted confidence in bin b, respectively and let N be the total number of variants.


(7) $$ECE=\sum_{b=1}^{M}\frac{n_{b}}{N}\times |{\text{conf}}_{b}-{\text{freq}}_{b}|$$

An ECE of 0 indicates perfect calibration, while higher values represent poorer calibration. Although ECE does not distinguish between overconfidence and underconfidence, these effects can be easily inferred from the accompanying reliability histograms.

#### Maximal Calibration Error.

Reported as the maximal calibration error across all bins with > 50 sample:

(8) $$MCE={\text{max}}_{b\in [M]\wedge \left|b_{i}\right|>50}\left\{|{\text{conf}}_{b}-{\text{freq}}_{b}|\right\}$$

### P-value and multiple hypothesis correction.

For Figure 1a, p-values were computed using Fisher’s exact test and adjusted for multiple comparisons with the Benjamini-Hochberg procedure. For the MI analysis (Figure 2b) p-values were calculated using the Mann-Whitney U test (SciPy’s *mannwhitneyu,* two-sided test) over 1,000 non-parametric bootstrap MI-values per attribute at the residue vs the protein level. The p-value reported in Figure 2b and Figure S3 corresponds to the default two-tailed p-value from SciPy’s *person* method, calculated based on the t-distribution.

### The RaCoon Pipeline

#### Partitioning and Pruning.

To construct the calibration tree, we first build a complete tree over all chosen binary residue attributes, where each leaf represents a distinct attribute combination. Because sparse attribute subsets are later pruned, the order of partitioning is important. We begin by dividing the data into residues from short and long proteins, and then iteratively split each node using the attribute that shows the most significant class-conditional feature distribution difference within that subset until all attributes have been exhausted (Algorithm 1, Methods). This greedy ordering prioritizes the most discriminative attributes near the root and facilitates subsequent pruning by grouping nodes in ascending order of conditional distribution difference. After the initial tree is constructed, we iteratively prune leaves with insufficient data until all remaining leaves contain sufficient samples (Algorithm 2, Methods). Following hyperparameters tuning we set a minimum threshold of 1600 variants per node, requiring at least 400 pathogenic and 400 benign variants. This criterion ensures that each subgroup contains sufficient training data for fitting the GMMs and enough held-out data to justify separate calibration (Figure S8, Figure S9b, Methods).

**Algorithm 1:** Dataset partitioning

Define a list binary partitioning attributes # [is_disordered, is_sulfur_binding, in_ppi] Initialize a tree with a single root node containing the unpartitioned dataset Split the dataset at the root by protein length (short vs. long) Associate each new node with the list of possible partitioning properties Iterative partitioning for each node at the current depth: 5.1. If no partitioning attributes remain, stop splitting this node. 5.2. For each available attribute, compute the class-conditional feature distribution difference within the node’s data.^†^ 5.3. Split the node to the positive and negative selections of the attribute with the maximal distribution difference split_properties 5.4. remove the used attribute from each child’s list of available splits. Repeat Step 5 for each newly created node until all attributes have been exhausted or stopping criteria (e.g., minimum sample size) are met. return the calibration tree.

† estimated using Jensen-Shanon Divergence over the normalized entropy values of a subset of benign and pathogenic variants of the data

**Algorithm 2:** Pruning

Set $N,\;n_{b},\;n_{p}$ the minimal thresholds for the total number variants, benign variants, and pathogenic variants per leaf. Traverse over all tree leafs until no leafs have been pruned: 2.1. If a leaf does not meet minimal thresholds remove the leaf^†^ Return the pruned tree

† the leaf data is still found in it’s parent data

#### Modelling.

Our calibration strategy relies on estimating the pathogenic and benign LLR score distributions rather than using explicitly labeled samples. A natural approach for modeling these distributions is to fit two GMMs for each node of the calibration tree, one for pathogenic and one for benign variants (Algorithm 3, Methods). We first estimate the fraction of pathogenic variants within each node using 500 randomly sampled variants (Methods). To reduce training size, the same variants are also used for GMM fitting. Each GMM is trained on 400 pathogenic and 400 benign variants, which we find yields results surpassing those obtained with tens of thousands of labeled examples (Figure S9a–b). Synthetic samples generated from the fitted GMMs, sampled according to the estimated pathogenic fraction, are then used to construct calibration histograms that map LLR values to pathogenicity probabilities.

The GMM-based strategy offers four key advantages. First, it effectively decouples calibration from the size of the node’s dataset, with only ~800 samples needed to fit the model (Figure S9a–b). Second, our hyperparameter choices intentionally constrain the expressibility of the GMMs (Methods), preventing them from overfitting the true data, capturing noise and outliers. Third, as generative models, GMMs enable unlimited synthetic sampling, facilitating the construction of finer-grained calibration histograms and thus improving scores interpretability. Importantly, drawing additional samples may, at most, lead to overfitting of the GMM distribution. Finally, drawing from GMMs prevents the calibration process from direct exposure to labeled examples, maintaining minimal downstream supervision and avoiding data leakage. In fact, even when GMMs are trained on the full node dataset, intentionally introducing potential leakage, no measurable improvement in AUROC is observed (Results-Ablations).

**Algorithm 3:** Fitting GMMs

Set $N_{GMM},\;N_{p}$ number of variants required to train a GMM and to estimate pathogenic fraction per node. Remove extreme outliers from the entire dataset using the modified z-score criterion (Methods). For each node in the calibration tree: 3.1. Randomly sample $N_{p}$ mutations from the node’s data and compute the pathogenic variants fraction. 3.2. Sample $N_{GMM}$ pathogenic and benign variants for model fitting^†^. 3.3. Train GMMs for the pathogenic and benign subsets. Return the GMMs and pathogenic fraction per-node.

† We reuse variants used for 3.1 and randomly sample additional variants to complete to $N_{GMM}$

#### Binning.

To translate LLR scores into meaningful pathogenicity estimates, we construct calibration histograms with equal-frequency bins where each bin represents a specific range of LLR values (Algorithm 4, Methods). As LLR scores are not uniformly distributed, using equal-frequency binning inherently adjusts bins granularity to match the underlying data density. Each bin is then assigned a pathogenic ratio score given by the empirical proportion of pathogenic variants within that bin. In practice, we draw 40,000 synthetic samples from the fitted GMMs, sampling from the pathogenic and benign components according to the estimated pathogenic to benign ratio in the node’s data. We perform an extensive hyperparameter search over the number of bins and synthetic samples, and find that 40,000 synthetic samples and 50 bins yield optimal AUROC performance while producing finely grained and interpretable scores (Figure S9c–d, Methods).

**Algorithm 4:** Binning

Set $N_{\text{bins}},\;N_{\text{sample}},\;P_{i}$ number of bins, number of synthetically drawn samples and the estimated pathogenic fraction per node respectively. For each node^†^ i in the calibration tree: 2.1. Draw synthetic samples from the fitted GMMs,using the following rule: 2.1.1. ${N_{\text{sample}}}^{*}P_{i}$ from the pathogenic GMM. 2.1.2. ${N_{\text{sample}}}^{*}(1-P_{i})$ from the benign GMM. Partition all sampled values into $N_{\text{bins}}$ equal-frequency bins. For each bin: 2.3.1. Compute the pathogenic frequency within the bin. Return the per-node histogram.

† In practise we calibrate all leaves, nodes with a single child and nodes corresponding to attributes without full coverage (such as PPIs) (Figure S8)

#### Mapping.

At inference time, a new variant is first assigned to its corresponding node in the calibration tree. Its raw LLR score is then mapped to the appropriate bin, from which the corresponding pathogenic fraction is retrieved (Algorithm 5, Methods). This procedure produces calibrated, interpretable predictions that directly translate model outputs into clinically meaningful frequencies.

**Algorithm-5:** Mapping:

Given a variant v and its associated attributes $A_{v}$, traverse the calibration tree until: 1.1. A leaf node is reached. 1.2. The next node in the path is missing^†^. 1.3. The next split involves a feature missing from $A_{v}$ (NaN). Retrieve the calibration histogram associated with the node.. Map the variant’s ESM1b LLR score to the appropriate histogram bin. Return the pathogenic fraction associated with that bin..

† The node was pruned

### Hyperparameters Tuning

#### GMM training sample size.

To isolate the effect of training set size on calibration performance we trained pathogenic and benign GMMs per-node with an increasing number of samples. We used a simplified calibration tree, partitioning only on length and fold, both having 100% coverage and proven to improve AUROC on ESM1b throughout the study. This step was essential because changing the number of training samples can also alter the pruning outcome, confounding the effects of tree composition and training set size on overall performance. To construct calibration histograms we fixed the number of synthetically drawn samples at 40,000 and the number of bins at 50. Reported results were obtained using the ClinVar_HQ dataset and represent the average over 100 independent training iterations, each consisting of 100 non-parametric bootstrap test iterations. Global AUROC was computed on an unseen test set composed of the remaining samples. We also report the effect of training sample size on the number of calibration subgroups in the resulting calibration tree (Figure S9b). We set the Racoon’s threshold at 400 training samples per node as it achieved significantly superior AUROC over the naive model while maintaining a large tree size.

Next, we compared the GMM-based calibration to direct histogram binning using the same simplified tree structure and hyperparameters. We progressively bin an increasing number of raw LLR scores up to the limit of the smallest node in the calibration tree, and evaluated AUROC on the remaining unseen samples. Direct binning yielded inferior results than the GMM approach by a significant margin even when using thousands of training samples. Furthermore, sampling over 5,000 scores did not improve performance, and may have even reduced it (Figure S9a).

Finally, we tested whether a hybrid strategy combining direct binning with synthetic sampling could further improve calibration. Using the same setup, we trained per-node GMMs on 400 samples, generated 40,000 synthetic samples and progressively combined them with increasing numbers of raw scores to construct the calibration histograms. The hybrid strategy only hindered the performance of the pure GMM approach, demonstrating degrading results with increased raw samples size (Figure S9a,b). We hypothesize that without incorporating denoising procedures, raw samples have a low signal to noise ratio requiring very large train size to reach comparable performance.

#### Estimation of pathogenic frequency.

To estimate the pathogenic frequency, we used 500 randomly sampled labels, which could also serve for GMM training to maintain a minimal training size. The labels were modeled as Bernoulli-distributed variables (1 pathogenic, 0 benign), and the sample size was selected to ensure an estimated pathogenic frequency p achieves a 95% confidence interval within ±5% margin. Under the normal approximation, a rigorous estimation, without assuming any prior on p, and without finite-population correction requires n=385 samples, we increased this to 500 to provide a conservative margin.

#### GMM-generated samples and number of bins.

Having established the minimum number of variants required for GMM training and the superiority of synthetic sampling over direct binning, we next optimized the number of synthetic samples drawn from the GMMs and the number of bins used in the calibration histograms. We first computed the full calibration tree on the ClinVar_HQ dataset using a minimum of 1600 total variants per node and the established thresholds of 400 benign and 400 pathogenic variants (Algorithms 1–3). All experiments were conducted over 100 independent GMM training iterations, each averaged over 100 non-parametric bootstrap test iterations.

To determine the optimal number of bins, we drew 100,000 synthetic samples from the GMMs and constructed calibration histograms with an increasing number of equal-frequency bins (Figure S9c). Drawing 100,000 samples ensured sufficient coverage without constraining bin granularity. Performance plateaued at approximately 40–50 bins, with no further improvement observed beyond this range. Increasing the number of bins above 50 may create an artificial impression of better calibration, as the apparent gain reflects over-splitting of existing bins rather than genuinely improved estimation of fine-grained frequencies. We therefore set the number of bins to 50, this provides a high resolution that accurately reflects the model’s learned distributions.

Next, we fixed the number of bins at 50 and analyzed AUROC as a function of the number of synthetic samples drawn from the GMMs (Figure S9d). Following Algorithm 4, samples were drawn according to the pathogenic-to-benign ratio of each node’s fitted GMMs. We found that performance plateaued at 40,000 synthetic samples per leaf. Fewer samples likely failed to estimate per-bin pathogenic probabilities accurately, given the relatively high number of bins, while larger sample sizes neither improved nor degraded performance. This result supports our earlier claim that while the calibration process can overfit the learned GMM distribution, the GMM itself acts as a regularizer, preventing overfitting to the raw LLR distribution. Consequently, we set 40,000 synthetic samples per node as the default for all subsequent experiments.

#### Immunity to data leakage.

To demonstrate that our pipeline can not memorize specific examples we compare RaCoon’s performance on a test-set consisting of samples seen during the calibration process and a test-set consisting of unseen samples. For each node, we retain an equal number of pathogenic and benign variants (matching the smaller class) and split this balanced dataset into balanced train and test subsets. The train set is used to fit the per-node GMMs. We then compare RaCoon’s performance on unseen variants (the test set) vs seen variants used to fit the GMMs - introducing deliberate data leakage. This setting ensures that in both scenarios the size and label distribution of the evaluation sets are equal, preventing biases in performance evaluation.

On the ClinVar dataset, RaCoon achieves an AUROC of 0.917 ± 0.001 in both settings, while on ProteinGym the AUROC is 0.892 ± 0.002 without data leakage and 0.893 ± 0.002 with full data leakage. These results demonstrate that even under extreme 100% leakage, RaCoon’s performance does not improve, confirming that the model does not memorize calibration samples. These AUROC values differ from those reported in the main text because this experiment uses very different test sets,designed specifically to equalize class distributions across all nodes.

### RaCoon server version

RaCoon is made publicly available through an open-access server. The server version returns predictions averaged over the median of 75 out of 100 randomly initialized RaCoon models trained on the ClinVar_HQ dataset, allowing standard deviations to be reported for each prediction. In addition, node-specific ECE and MCE values are provided. Support for node-specific classification thresholds will be incorporated in a future release.

## Supplementary Material

Supplement 1

Supplement 2

Supplement 3

Supplement 4

Supplement 5

## Figures and Tables

**Table 1.** Pathogenic variants rate shifts in ClinVar_HQ and ProteinGym.

Residue Attribute	Pathogenic Rate		Pathogenic Variants		Benign Variants		p-val^a^	
Dataset	ClinVar	ProteinGym	ClinVar	ProteinGym	ClinVar	ProteinGym	ClinVar	ProteinGym
	0.31	0.51	52,634	32,000	118,562	30,727	Baseline	
Disordered	0.10	0.19	5,700	3,038	48,845	12,742	$p\ll 1.0e^{-23}$	$p\ll 1.0e^{-23}$
Low homology^b^	0.19	0.42	2,361	1,503	10,108	2,068	$p\ll 1.0e^{-23}$	$p\ll 1.0e^{-23}$
Hydrophobic	0.28	0.48	16,173	9,419	40,674	10,287	$p<1.0e^{-23}$	$p<1.0e^{-14}$
Charged	0.30	0.52	15,124	9,857	35,660	9,260	$p<1.0e^{-7}$	p=0.19
Polar	0.37	0.57	20,388	12,004	34,797	8,907	$p\ll 1.0e^{-23}$	$p\ll 1.0e^{-23}$
Ordered	0.40	0.62	46,934	28,962	69,717	17,985	$p\ll 1.0e^{-23}$	$p\ll 1.0e^{-23}$
PPI	0.61	0.73	6,931	4,376	4,445	1,659	$p\ll 1.0e^{-23}$	$p\ll 1.0e^{-23}$

ProteinGym - clinical substitution benchmark ^a^multiple hypothesis correction applied (FDR), capped at ${1.0e}^{-23}$^b^sequences with Uniref90 cluster size < 10 (Methods).

**Table 2.** Summary of dataset used in the study.

Dataset Name	Total variants	Pathogenic variants	Benign variants	Unique sequences	Details
ClinVar_HQ	171,196	52,634	118,562	14,564	Curated directly from ClinVar
Well-annotated ClinVar genes	86,211	42,056	44,155	1,285	Sequences with at least 10 variants of which at least 4 pathogenic and 4 benign variants
ClinVar_BM	14,054	11,757	2,296	6,599	variants published in clinvar after may 2021, protein with unbalanced annotations excluded^9^
ClinVar_BM	56,368	18,765	37,603	6,272	Published by Cheng et al.^6^ proteins with no matching transcript excluded
ProteinGym	62,727	32,000	30,727	2,525	ProteinGym zero-shot clinical substitution benchmark^8^ obtained in June 2025

**Table 3.** List of VEP benchmarked in the study.

Model Name	Supervision Type	Features Used^a^	Train Data (Supervised)	Bounded Scores (0–1)	Reference
PrimateAI	Unsupervised	MSA, structural features	-	Yes	^101^
LIST-S2	Unsupervised	MSA	-	Yes	^102^
PROVEAN	Unsupervised	MSA	-	No	^103^
ESM1b	Unsupervised	Sequence only	-	No	^4^
SIFT	Unsupervised	MSA	-	Yes	^104^
MutationAssessor	Unsupervised	MSA	-	No	^105^
ESM1v	Unsupervised	Sequence only	-	No	^21^
EVE	Unsupervised	MSA	-	No^d^	^1^
AlphaMissense	Semi-Supervised	MSA, structural features	ClinVar (Calibration)	Yes	^6^
FATHMM	Supervised	MSA	HGMD + UniProt/SwissProt	No	^106^
DANN	Supervised	MSA, genomic features	Allele frequency^e^ (1000 Genomes Project)	Yes	^107^
MPC	Supervised	Metapredictor, residue features	Population frequency (ExAC)	No	^108^
DEOGEN2	Supervised	MSA, structural gene, residue and pathway features	Humsavar	Yes	^109^
PolyPhen-2 (HDIV)	Supervised	MSA, structural and residue features	HumDiv	Yes	^110^
PolyPhen-2 (HVAR)	Supervised	MSA, structural and residue features	HumVar	Yes	
CADD	Supervised	MSA, genomic features	Population frequency^e^ (1000 Genomes Project)	No^b^	^111^
gMVP	Supervised	MSA, structural and genomic features	ClinVar + Population frequency (gnomAD)	Yes	^112^
REVEL	Supervised	Metapredictor	HGMD and Population frequency (1000 Genomes/ESP cohorts)	Yes	^113^
BayesDel (noAF)	Supervised	Metapredictor	ClinVar, UniProt Population frequency^f^	No^c^	^114^
BayesDel (addAF)	Supervised	Metapredictor	ClinVar, UniProt Population frequency^f^	No^c^	
VEST4	Supervised	MSA, structural, residue and genomic features	HGMD, ClinVar, Population frequency (Exome Sequencing Project/1000 Genomes)	Yes	^115^
ClinPred	Supervised	Metapredictor	ClinVar	Yes	^10^
MetaRNN	Supervised	Metapredictor	ClinVar	Yes	^11^

HDIV - trained on HumDiv, HVAR- trained on Humvar, noAF and addAF whether allele frequency is used, HGMD - Human Gene Mutation Database, ExAC - Exome Aggregation Consortium. Metapredictors employ multiple classifiers with various features.

a MSA also includes models trained on various homology models,

b also report the scaled PHRED score which we do not use,

c scores scaled around 0,

d also report the scaled EVE-score used in Figure 3c.

e variants not observed in humans defined as pathogenic, common variants defined as benign.

f UniProt polymorphisms, dbSNP, 1000 Genomes, ExAC, and UK10K cohorts

**Table 4.** Summary of protein and residue properties.

Attribute	Predictor	Threshold / Condition	Available Predictions		Positive Predictions^a^	
			ClinVar_HQ (N=171,196)	ProteinGym (N=62,727)	ClinVar_HQ	ProteinGym
Disordered	ALBATROSS Metapredict v1.3^44,119^	0.7	100%	100%	31.7%	25.1%
PPI	PIONEER^73^	Binary	74%	77%	9%	12%
Homology (low)	UniRef90^122,123^	10	89.7%	88.3%	8.5%	6.8%
Length (long)	sequence	1022	100%	100%	47.1%	38.0%
Polar	Residue	S, T, C, Y, N, Q, G	100%	100%	32.3%	33.3%
Hydrophobic	Residue	A, V, I, L, M, F, Y, W	100%	100%	33.2%	31.4%
Charged	Residue	R, H, K, D, E	100%	100%	29.6%	30.5%
Aromatic	Residue	F, W, Y, H	100%	100%	6.8%	7.7%
Acidic	Residue	D, E	100%	100%	8.8%	9.4%
Basic	Residue	R, H, K	100%	100%	20.7%	21.1%
Uncharged Polar	Residue	S, T, N, Q, C, Y	100%	100%	22.8%	21.9%
Small	Residue	G, A, S, T, P	100%	100%	36.9%	37.5%
Sulfur Binding	Residue	C, M	100%	100%	6.8%	5.9%
Proline or Glycine	Residue	P, G	100%	100%	16.3%	18.5%
Aliphatic	Residue	V, I, L, M, A	100%	100%	28.9%	26.2%
Helix Breaker	Residue	G, P	100%	100%	16.3%	18.5%
Beta Branched	Residue	I, V, T	100%	100%	17.6%	15.3%

a percent of available prediction.

**Table 5.** Mutual Information at the protein and residue level.

Residue attribute	Residue-level MI		Protein-level MI		p-value
	mean	std	mean	std	
Disorder level	0.014	6.16× 10^−3^	0.051	6.88× 10^−4^	$p\ll 1.0\times 10^{-22}$
Homology count	0.005	4.73× 10^−3^	0.014	4.75× 10^−4^	p=0.21
Interface status	0.016	6.39× 10^−3^	0.004	2.00× 10^−4^	p=0.99

p-value calculated using Mann–Whitney U-test (two sided) for residue-level MI > protein-level MI.

**Table 6.** Bin size effect on JSD calculation.

Bin-size	Kendall τ	p-value
200 vs 100	0.857	$p<1.0e^{-12}$
20 vs 50	0.784	$p<1.0e^{-10}$
200 vs 25	0.721	$p<1.0e^{-9}$
100 vs 50	0.921	$p<1.0e^{-14}$
10 vs 25	0.851	* $p<1.0e^{-12}$ *
50 vs 25	0.917	* $p<1.0e^{-15}$ *

p-value two-sided test against τ=0.

## Data Availability

All datasets used in the study are available at zenodo.org (17690936).

## Code availability

All code used to implement RaCoon is publicly available through the RaCoon Github repository.
