# Supplementary material for: Calibrated Variant Effect Prediction at the Residue Level Using Conditional Score Distributions
Source: bioRxiv. 2025 Nov 26:2025.11.24.690189. Preprint. [Version 1] doi: 10.1101/2025.11.24.690189 (PMC12697679; doi:10.1101/2025.11.24.690189)
Supplement: Supplement 1 [file media-1.pdf]

Figure S1. LLR score distributions in ClinVar and ProteinGym

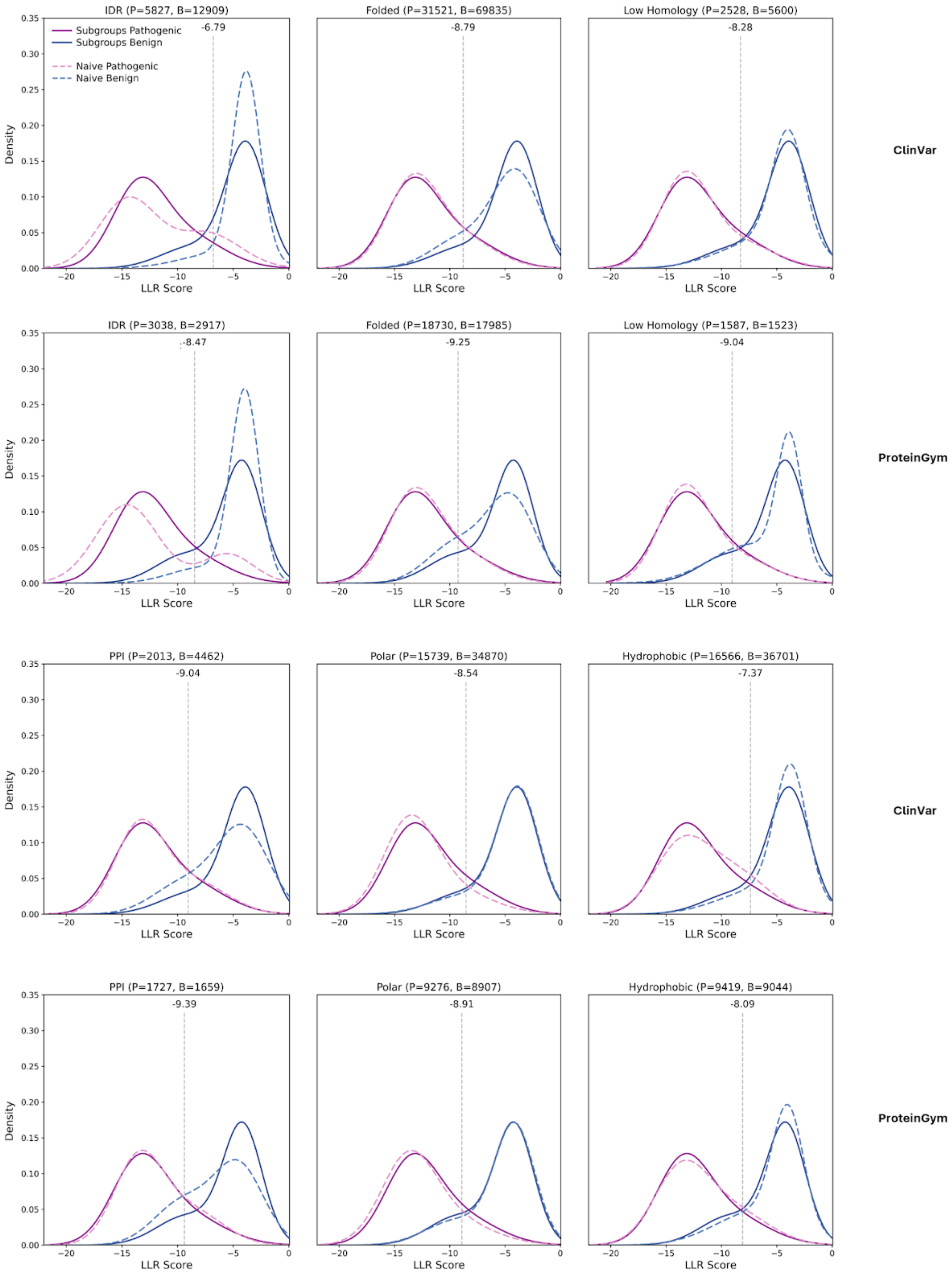

Figure 1S: LLR distribution of selected attributes. Distribution of LLR scores for pathogenic and benign variants in ClinVar_HQ and ProteinGym datasets. Dotted purple and blue curves - pathogenic and benign GMMs distribution over the entire dataset. Solid curves - subgroup specific GMMs distributions. *P*, *B* denote the number of pathogenic and benign variants per subgroup. Dotted line - optimal classification thresholds (J-statistic) Distributions fitted using two-component GMMs.

Figure S2. Entropy distribution differences with different scoring stratagies

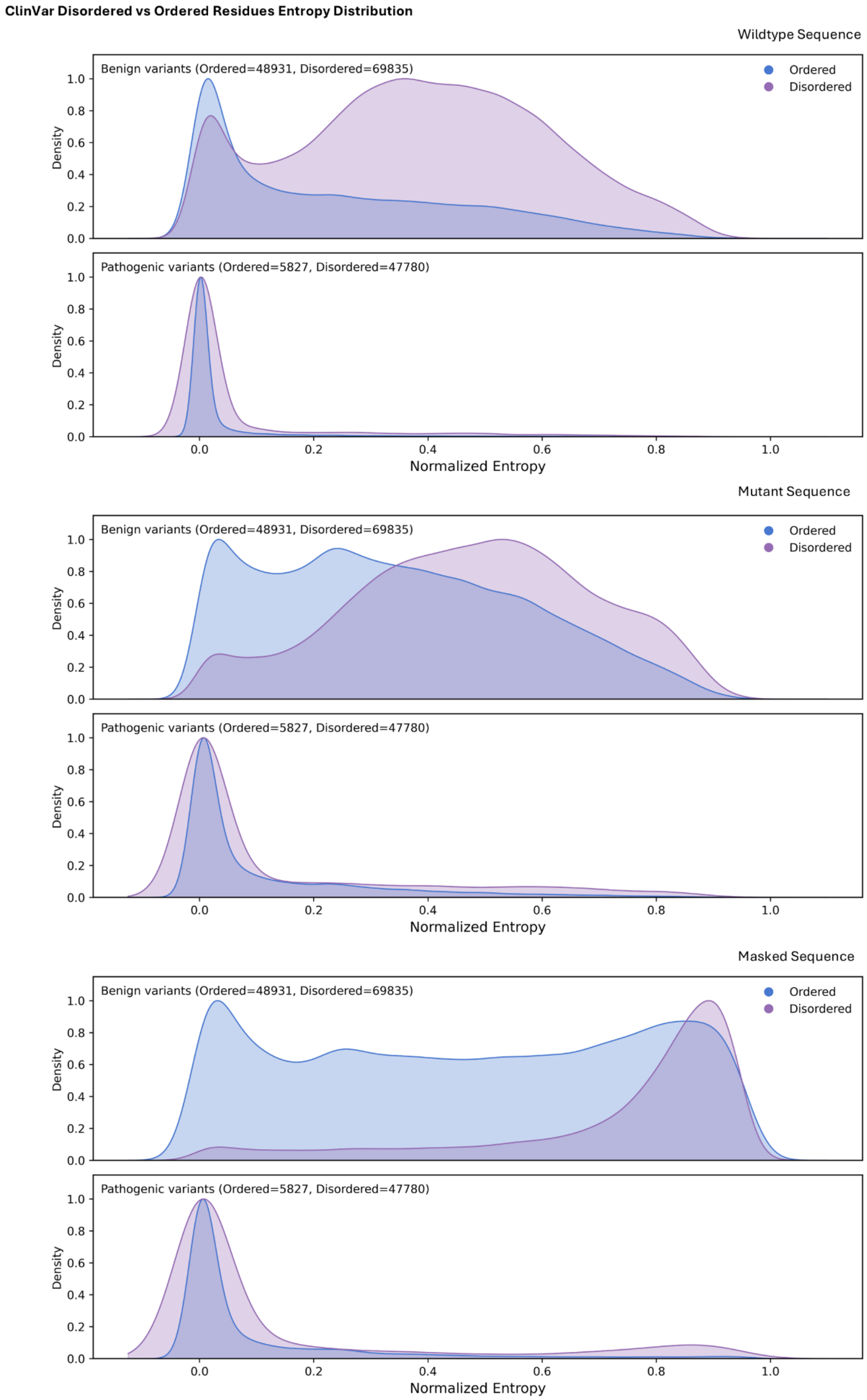

ClinVar Polar vs non-Polar Residues Entropy Distribution

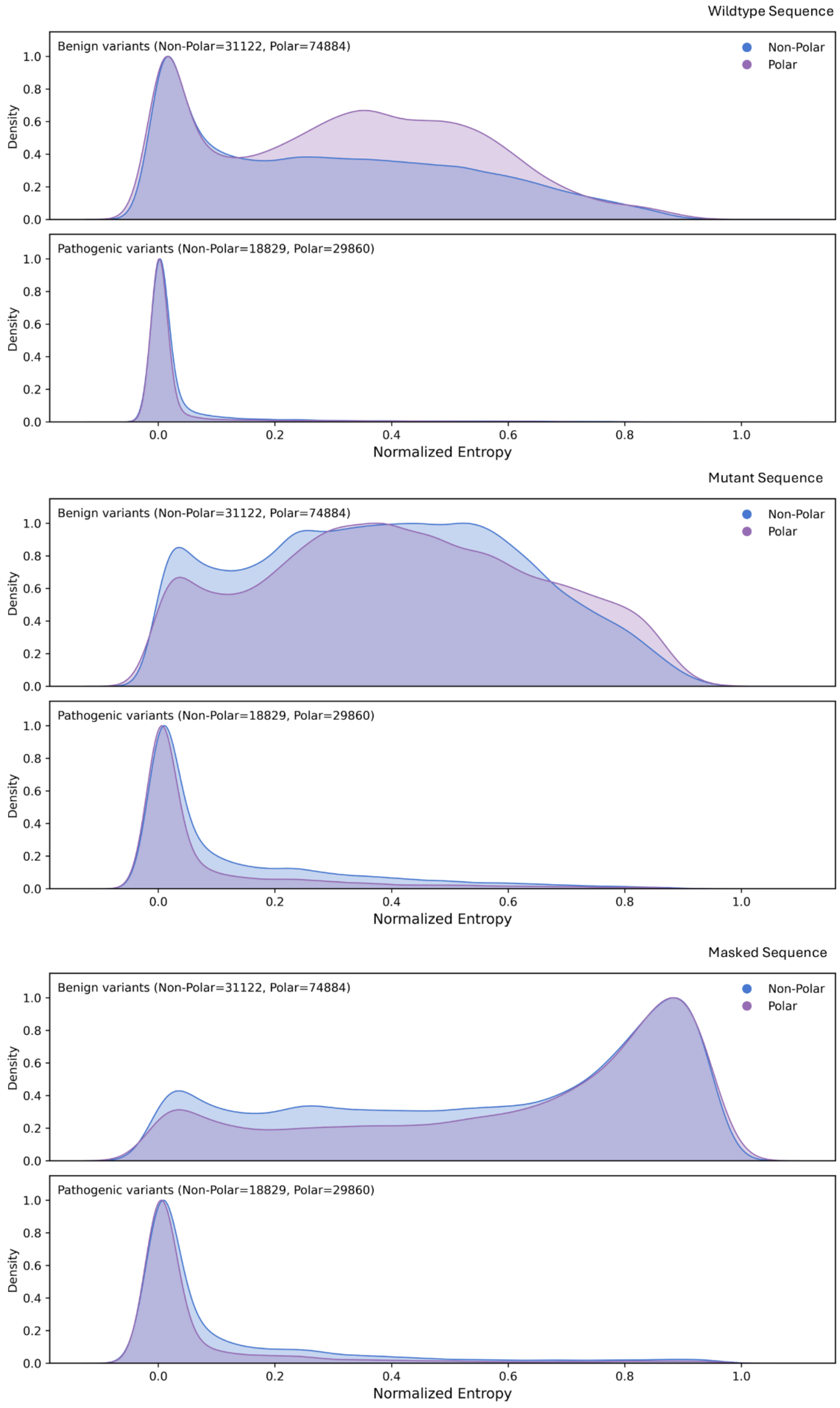

ClinVar Interface vs non-Interface Residues Entropy Distribution

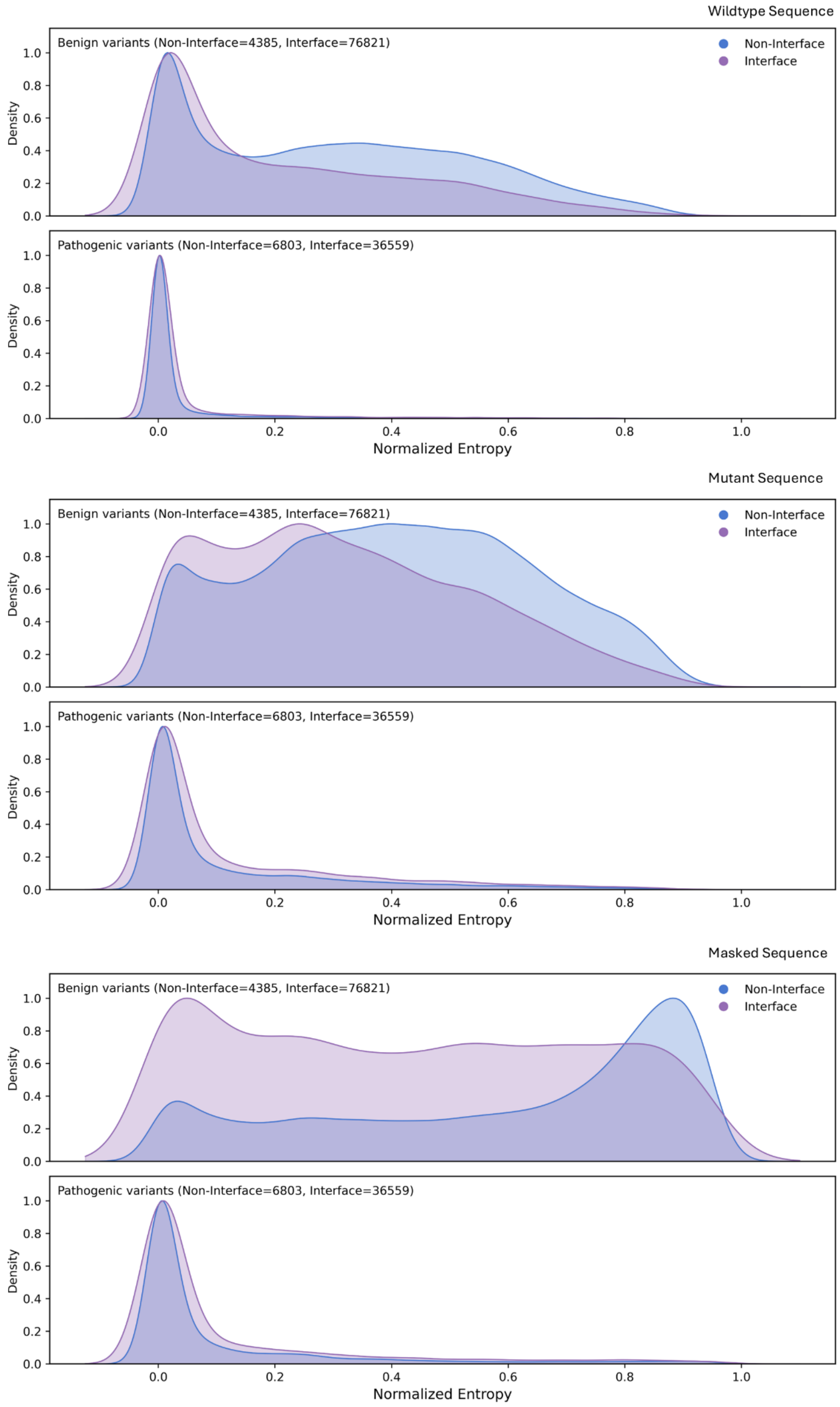

Figure S2. Entropy distributions with different representation strategies. Conditional distributions of flipped normalized entropy (1 = complete uncertainty, 0 = complete certainty) for disordered vs. ordered (**a**), polar vs. non-polar (**b**), and interface vs. non-interface (**c**) residues. Each figure shows wild-type (top), mutant (middle), and masked (bottom) input sequence, and grouped by benign variants (top) and pathogenic variants (bottom).

Figure S3. Relative (per sample) JSD contribution to AUC

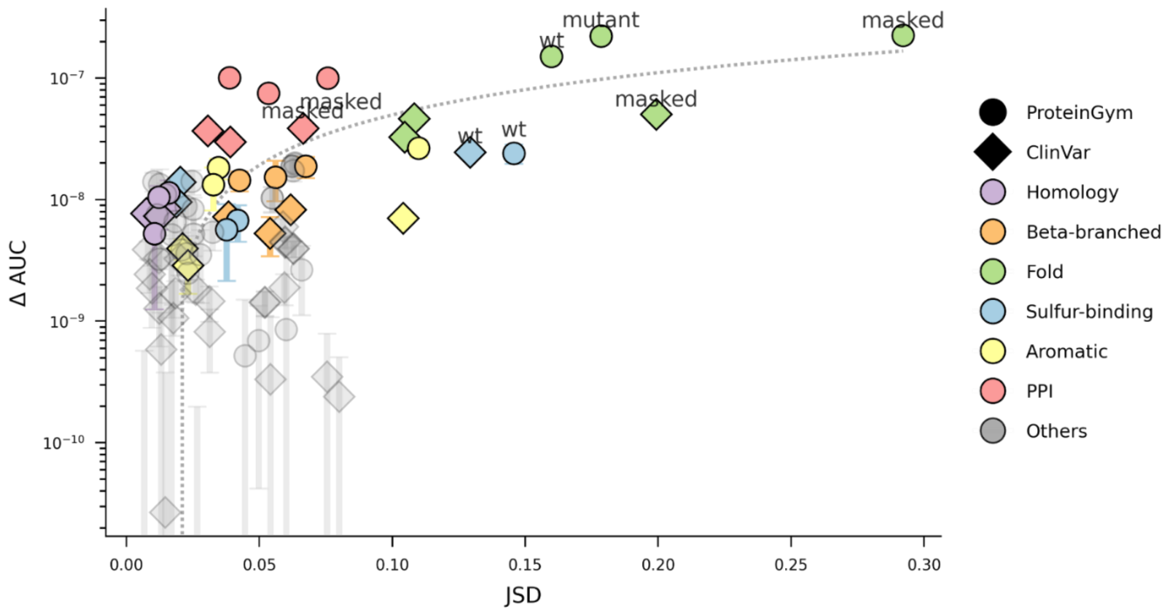

Figure S3. Relative contribution of class-conditional feature distribution divergence to AUROC gain (ESM1b). Change in overall AUROC normalized by the number of samples per subgroup (minimum count between positive and negative selections). y-axis - normalized AUROC (relative to the uncalibrated model) as a function of class-conditional shift (x-axis; Jensen–Shannon divergence between attribute partitions). Pearson correlation r = 0.728, p-value < 1.0e-17 Calibration performed on one-third of the data, leaving two-thirds for testing (Methods). Error bars indicate ± 1 SD across three training folds and 100 non-parametric bootstrap test iterations per fold. Circles and diamonds correspond to the ProteinGym and ClinVar_HQ benchmarks, respectively; symbol color denotes the calibrated residue attribute (legend), and grey transparent symbols represent other tested attributes (Table S3).

Figure S4. Extended Thresholds Shifts

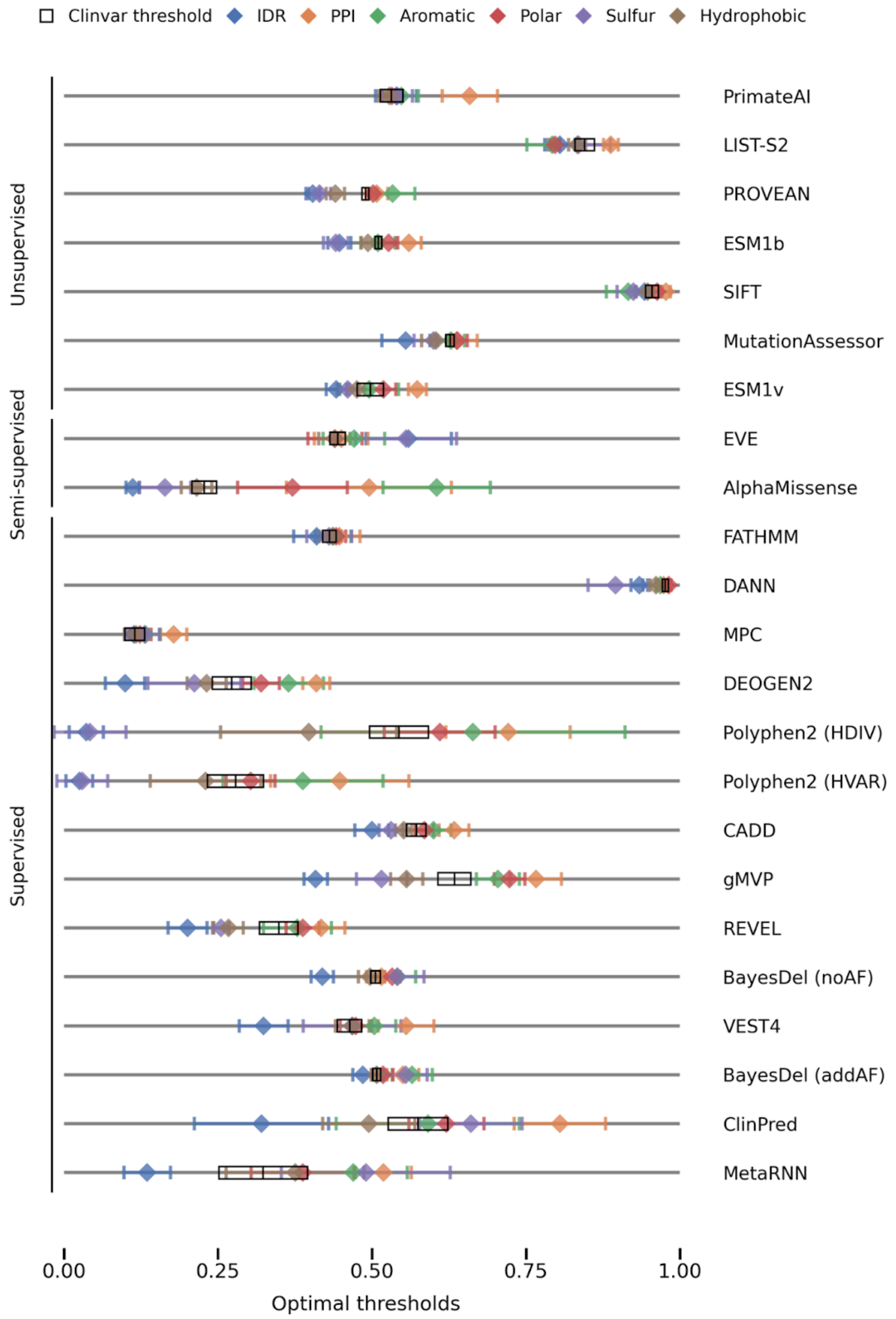

Figure S4: Optimal classification threshold shifts across VEPs (extended). Shifts in optimal discrimination thresholds (diamonds; maximal J-statistic) across normalized model scores in the ClinVar_BM dataset. Error bars indicate ±1 SD over 1,000 non-parametric bootstrap iterations. Black rectangle marks the naive threshold (mean ± SD) across the entire ClinVar_BM dataset.

Figure S5. Changes in AUROC

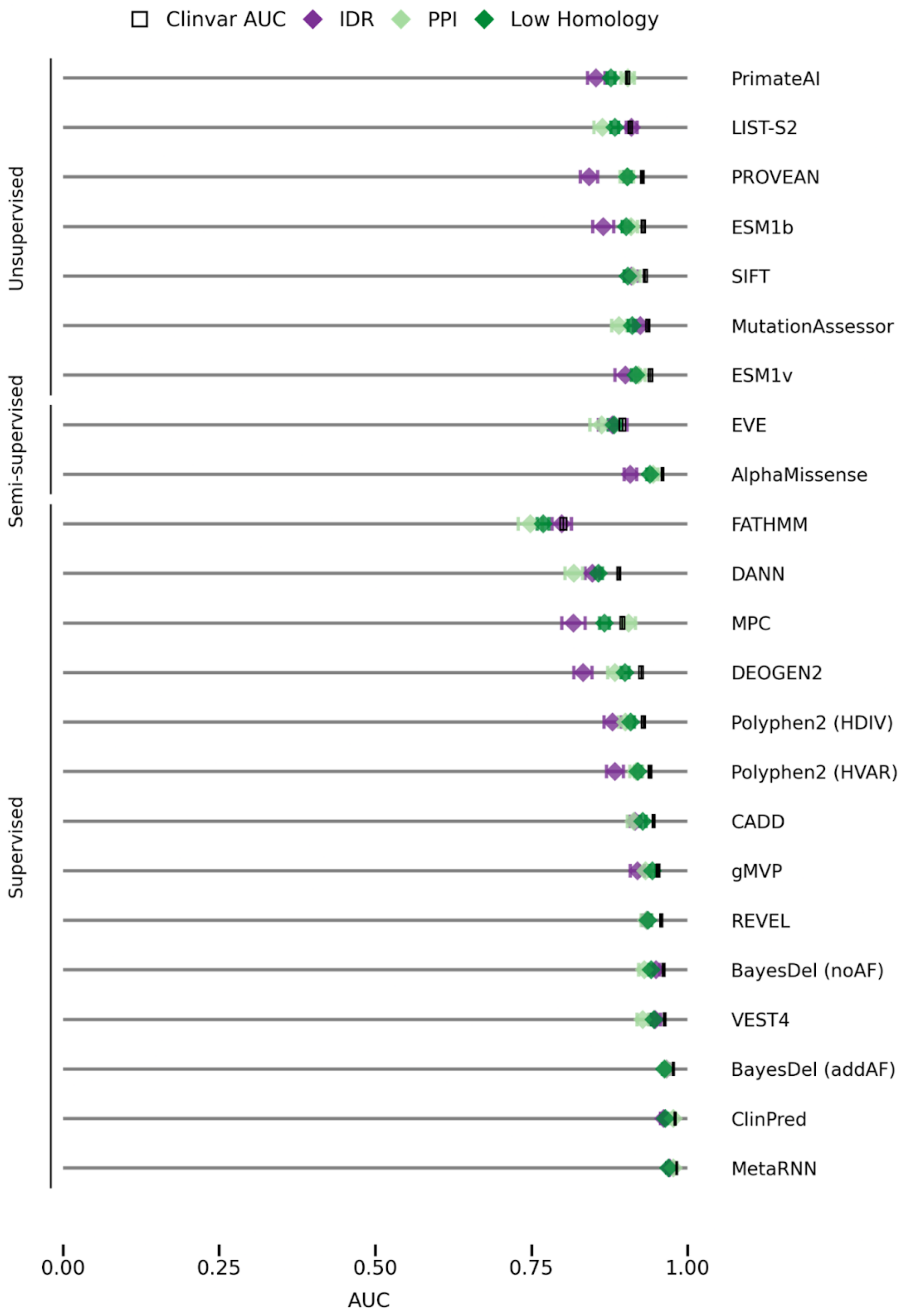

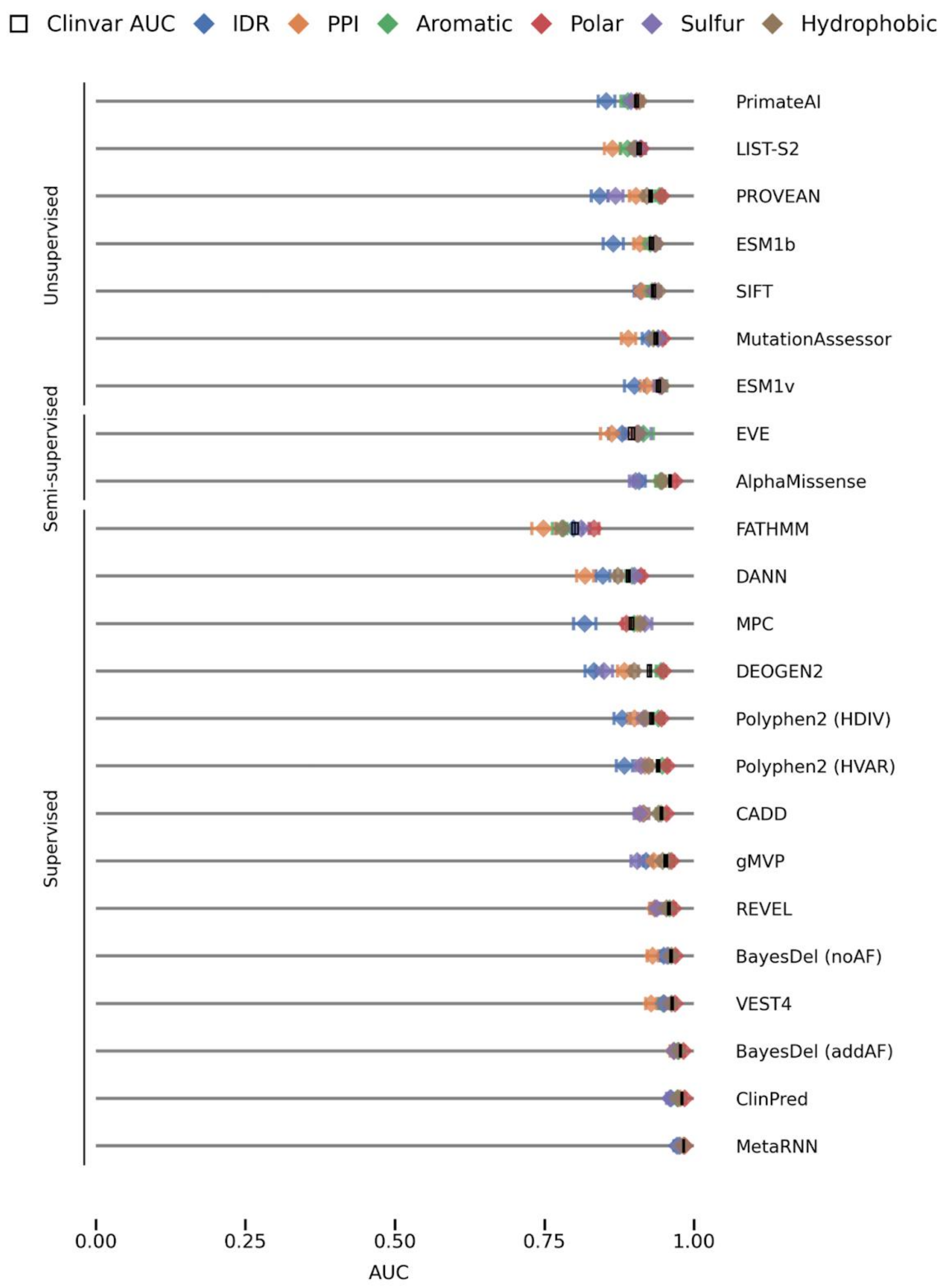

Figure S5. VEP performance across variant subgroups. Performance shifts shown as changes in AUROC (Diamonds) across all variants within each subgroup: (**a**) attributes showing the most significant shifts and (**b**) extended attributes set. Error bars indicate ± 1 SD over 1,000 non-parametric bootstrap iterations. Black rectangle marks the mean ± 1SD across the entire ClinVar_BM dataset.

Figure S6. Affect of residue-level regression on subgroup calibration

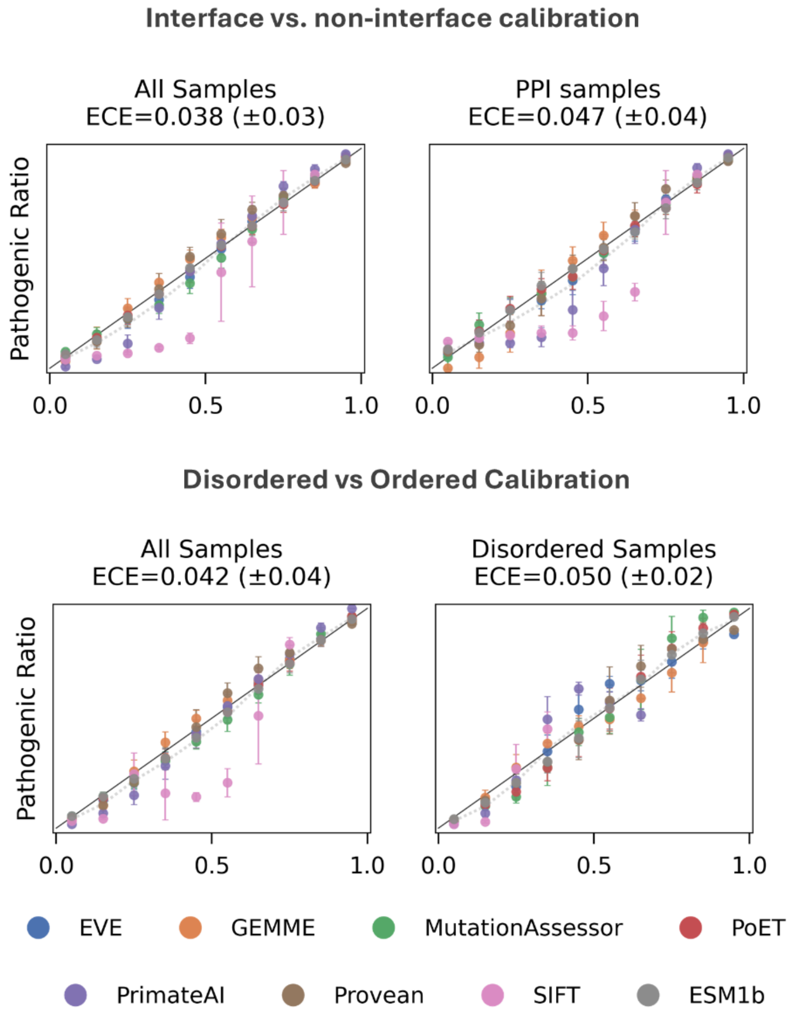

Figure S6. Affect of differential residue-level regression on subgroup calibration. Reliability histograms show predicted confidence (x-axis, 10 equal-width bins) versus observed pathogenic frequency (y-axis) following subgroup specific calibration (top, interface vs. non-interface, bottom, disordered vs. Ordered residues). Models calibrated by fitting separate logistic regression using 250 samples per subgroup (Methods). Perfect calibration follows y=x; dotted lines show the mean trend across models. Circles mark per-model bin frequencies; error bars indicate ±1 SD across 100 calibration iterations. Panels display the full ProteinGym dataset (left), PPI variants (top right), and disordered variants (bottom right). ECE = the mean weighted Expected Calibration Error across models

Figure S7. Affect or residue-specific on AUROC

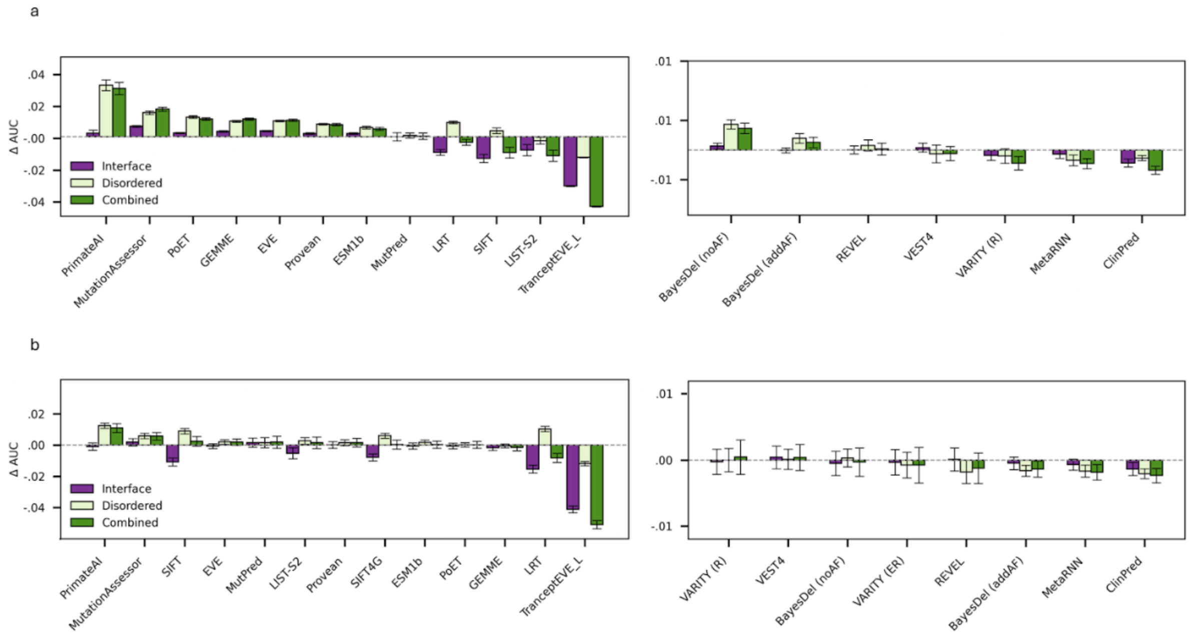

Figure S7. Effect of residue-specific calibration on AUROC. Shown as global ΔAUROC (**a**) and per-protein ΔAUROC (**b**) relative to baseline calibration over the entire dataset for unsupervised (left) and supervised (right) VEPs. Calibration performed using 250 samples per subgroup (Methods). Error bars indicate ±1 SD across 1,000 bootstrap iterations.

Figure S8. RaCoon’s Calibration Tree

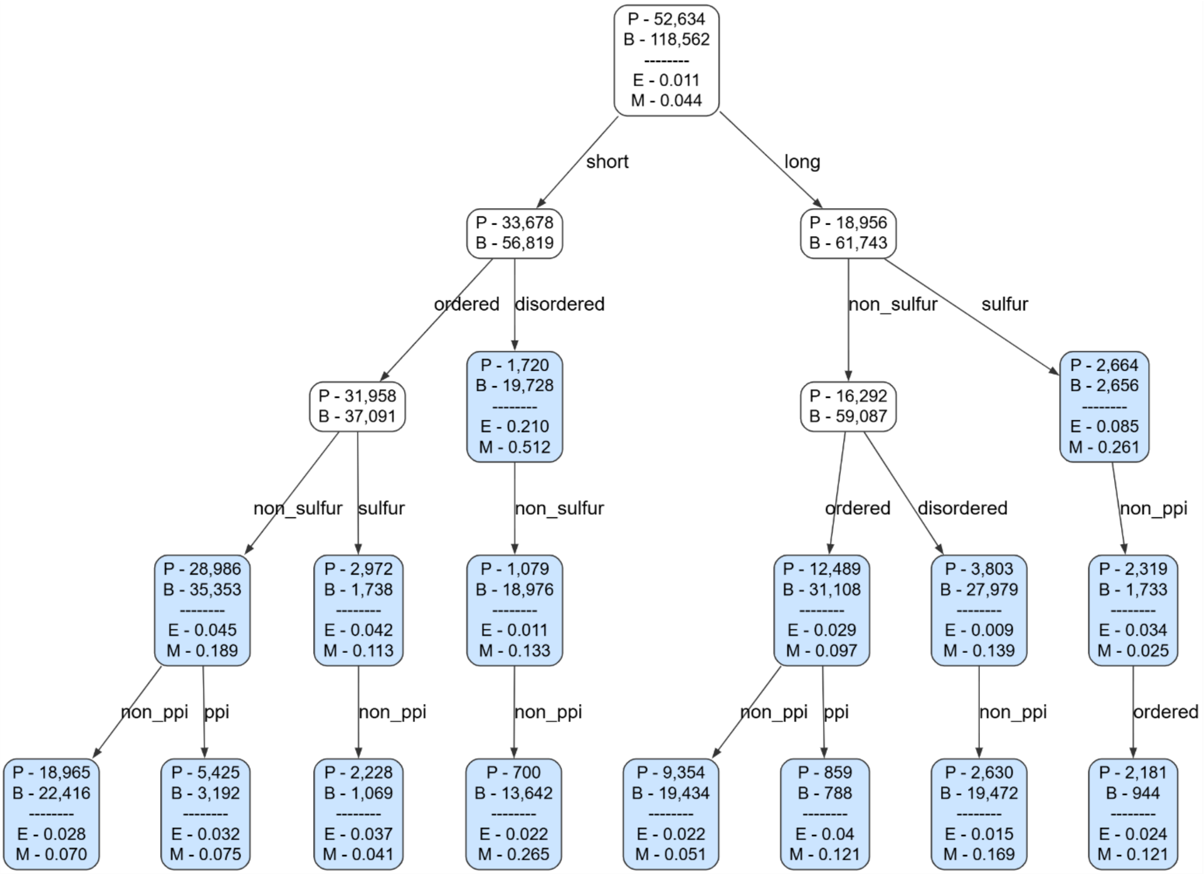

Figure S8. RaCoon calibration tree. Calibration tree obtained after partitioning and pruning of the ClinVar_HQ dataset (Methods, Algorithms 1-2). Node thresholds: ≥400 pathogenic, ≥400 benign, and ≥1,600 total variants. Blue nodes denote calibrated subgroups (all leaves, single-child nodes, and partially covered attributes). P and B - number of pathogenic and benign variants per (train and test included). E and M (ECE and MCE, respectively) - computed on the held-out test set per node.

Figure S9. Racoon Hyperparameters Search

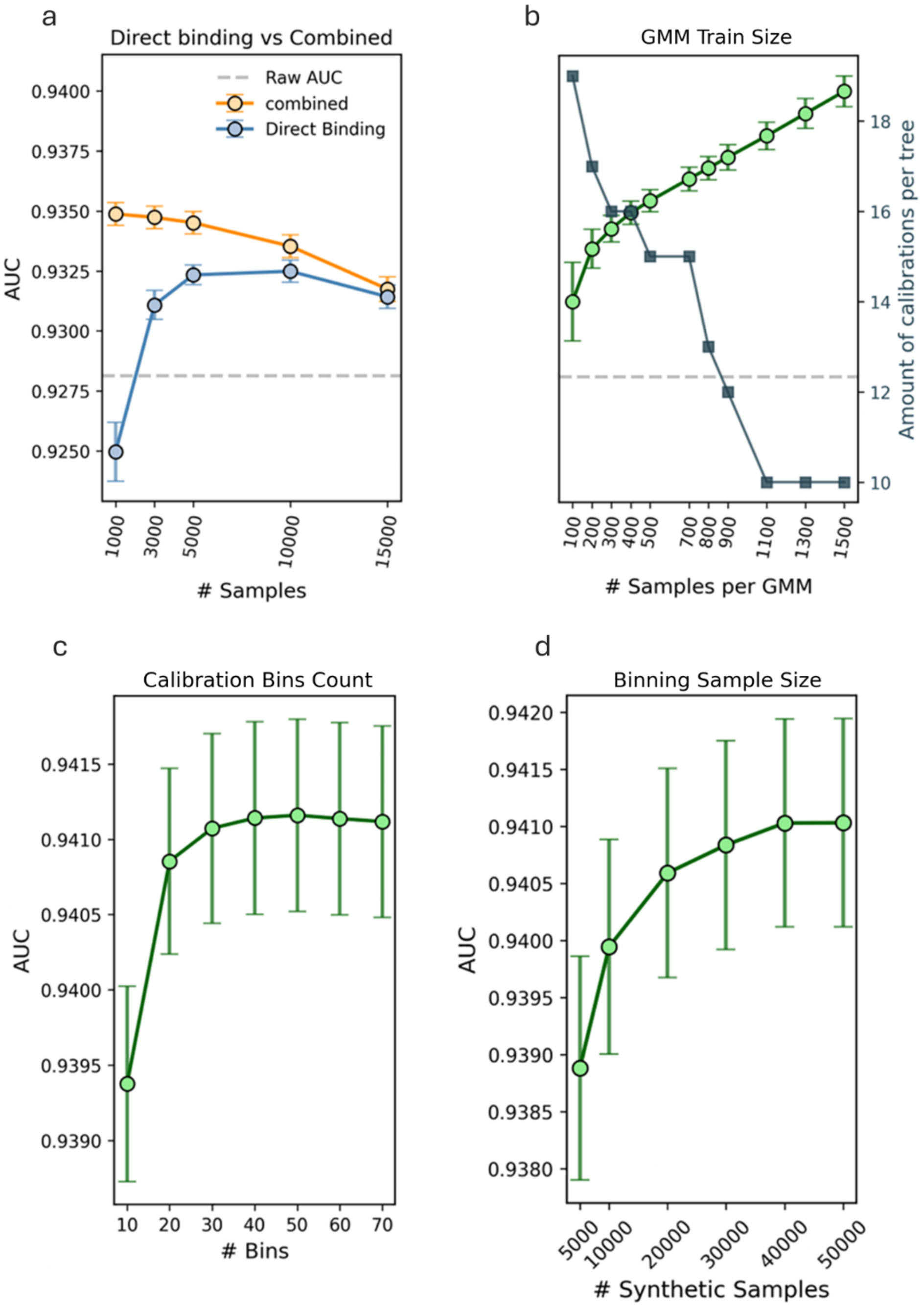

Figure S9. Ablation studies of the RaCoon pipeline. All results show changes in global AUROC on test samples not used during calibration. Error bars indicate ± 1 SD across 100 training (calibration) iterations, each averaged over 100 non-parametric bootstrap test iterations. **a.** Effect of binning method on AUROC: direct binning of raw LLR scores (blue) versus a hybrid method combining N = 40,000 synthetic samples from benign and pathogenic GMMs fitted to raw LLRs (orange). **b.** Effect of training sample size (per GMM) on AUROC (green) and on the number of unique calibrated subgroups retained after pruning (blue, right y-axis). **c.** Effect of the calibration histogram bins on AUROC using 100,000 synthetic samples from the benign and pathogenic GMMs. **d.** Effect of the number of synthetic GMMs samples on AUROC using 50 calibration bins.

Figure S10. RaCoon results on ClinVar and ProteinGym

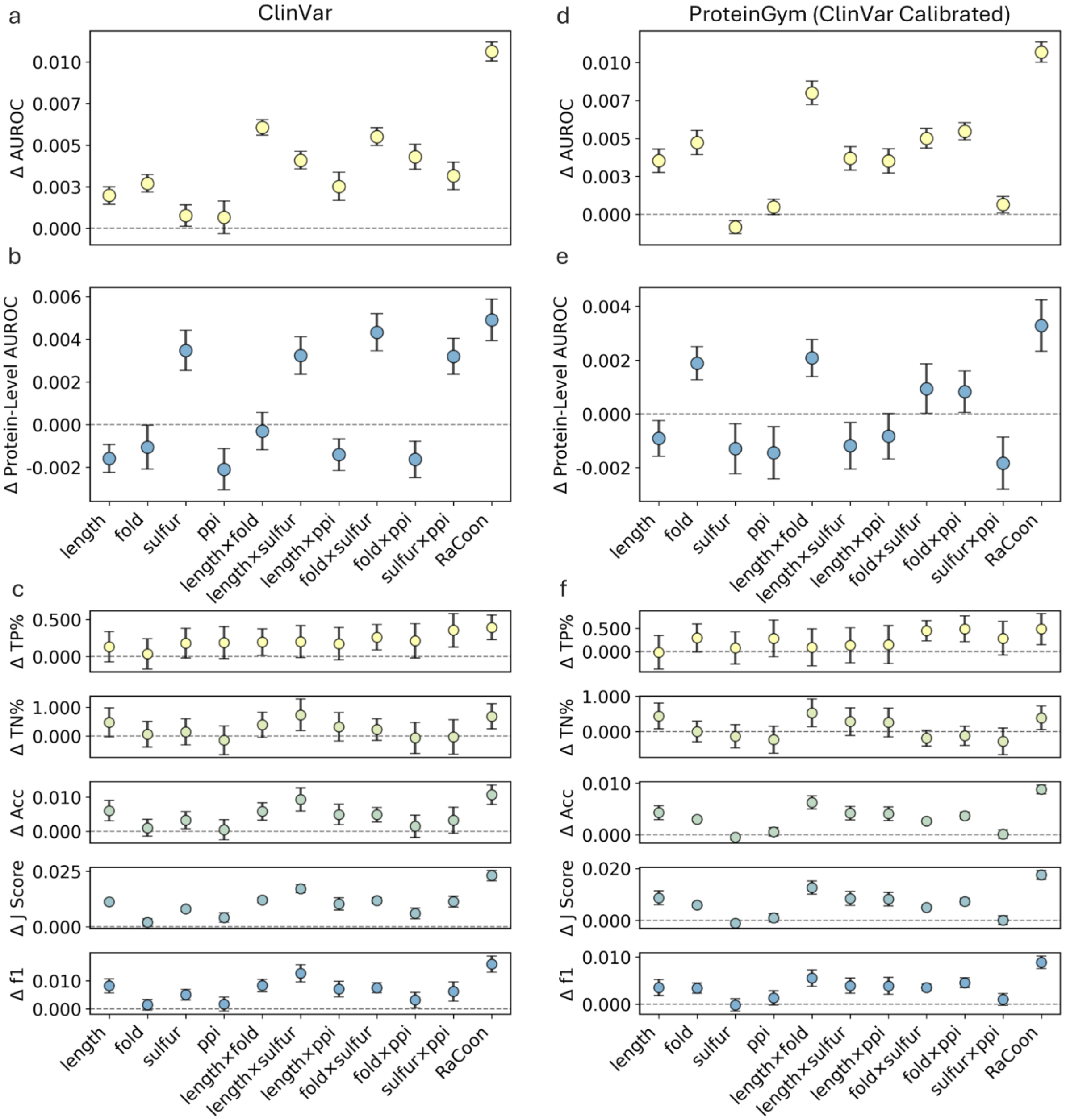

Figure S10. Performance evaluation on ClinVar and ProteinGym (ClinVar calibration). Changes in overall performance relative to the uncalibrated ESM1b. Error bars indicate ± 1 SD across 100 randomized training iterations, each evaluated with 100 non-parametric bootstrap test resamples. Top: global AUROC; middle: per-protein AUROC; bottom: discrimination metrics. Per-protein AUROC computed for sequences containing ≥ 10 variants with at least one pathogenic and one benign label. TP = true positive; TN = true negative; thresholds were optimized per model using the Youden J-statistic. **a-c** Evaluation and calibration on ClinVar_HQ **d-f** Evaluation on ProteinGym, calibration on ClinVar_HQ.

Figure S11. RaCoon ROC Curves

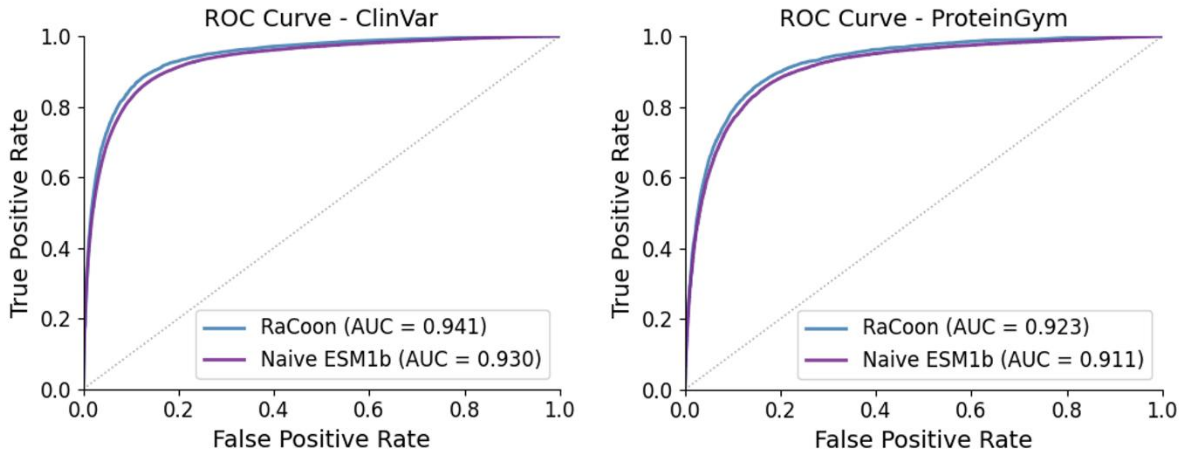

Figure S11. ROC Curve Racoon vs ESM1b. Performance evaluated on a test set of variants not used for RaCoon's calibration across the ClinVar_HQ dataset (pathogenic = 46804, benign = 112236) and on the ProteinGym clinical substitution benchmark (pathogenic = 27853, benign = 26073),
